# Implementation barriers of Brazil’s national home visitation program for early childhood development: A qualitative evaluation

**DOI:** 10.1371/journal.pgph.0005203

**Published:** 2026-04-10

**Authors:** Christopher M. Westgard, Ana Carolina Silva Onofre, Nayara Vieira Peres, Luana Bessa, Ana Luiza Raggio Colagrossi, Jaqueline Lima Santos, Diego Fontana Siqueira Cunha, Alexandra Brentani

**Affiliations:** 1 Harvard T. H. Chan School of Public Health, Harvard University, Boston, Massachusetts, United States of America; 2 Gillings School of Global Public Health, University of North Carolina at Chapel Hill, Chapel Hill, North Carolina, United States of America; 3 Department of Pediatrics, University of São Paulo Medical School, São Paulo, SP, Brazil; 4 Child Development Center, University of São Paulo Medical School CEDI-FMUSP, São Paulo, SP, Brazil; PLOS: Public Library of Science, UNITED STATES OF AMERICA

## Abstract

This research delves into the challenges faced in implementing home visitation programs for early childhood development, focusing on the Programa Criança Feliz (PCF) in Brazil. We aimed to better understand what barriers are hindering PCF from creating a greater positive impact on its primary outcomes. This is a cross-sectional qualitative study. We conducted qualitative interviews and focus group discussions in 11 Brazilian municipalities. The study participants were asked about expectations of the workforce, curriculum content, training, and supervision, working conditions, program design, favorable environment, and quality monitoring and assurance. Data analysis followed the Framework Method approach. The codebook followed the categorization from the Home Visiting Workforce Needs Assessment framework and the Consolidated Framework for Implementation Research framework. A total of 38 participants took part in the 6 focus group discussions and 11 key informant interviews. A range of challenges impeding PCF implementation were identified. The home visitation guidelines lack a clear structured curriculum for the home visitors to follow. Some home visitors felt overburdened due to a lack of training, limited materials, long commutes to visits, and a high number of families to visit. High turnover rates of home visitors further contribute to the difficulties. Hard-to-reach communities and families were often excluded from the program because the home visit quota goals do not consider travel time to distant communities. The cascading training structure of PCF often results in the home visitors being trained by individuals with little or no training in child development, pedagogy, or the program structure. Our findings reveal barriers created by the content and implementation of PCF, shedding light on the need for tailored strategies to overcome these challenges. These results provide practical, transferable insights for home visitation and analogous early-childhood programs operating in resource-constrained settings, with applicability to other contexts contingent on contextual similarity.

## Introduction

The importance of early childhood development is increasingly recognized and prioritized across the world, especially in low-resource settings. It is important to support child development in underserved communities because developmental delay has several long-term outcomes, including poor school performance, lower life-time earnings, high fertility rates, and poor health outcomes [1,2]. Several strategies have been developed to support early childhood development, such as community centers for infant stimulation, preschool programs, and home visits to support effective parenting [3,4].

A large body of evidence shows that home visits by community-based paraprofessionals (hereby referred to as home visitors) can be an effective strategy to improve early childhood development in impoverished and under-served communities, teaching caregivers how to better stimulate the child’s development and provide nurturing care [5–11]. Home visitors (HVs) can help caregivers develop more effective parental practices, creating improvements in parenting knowledge, practices, and parent-child interactions [3,8,11–17]. This has been shown to have positive effects on cognitive, language, psychosocial, and motor development [18].

Although HVs show great promise as a community-based education tool, outcomes vary greatly [19–21]. Many home visiting programs have been implemented with limited results, finding it difficult to achieve and sustain positive effects across diverse populations and over time [17,19,22,23] The impact of the programs is often hindered by implementation barriers that lead to reduced HV performance, inadequate support material, ineffective supervision and training structure, and poor quality improvement efforts [24,25].

The national home visitation program for early childhood development (ECD) in Brazil, Programa Criança Feliz (PCF), is the largest home visitation program to support child development in the world [26]. According to the Federal Government, until 2021, PCF has been implemented in 3085 out of the 5570 Brazilian municipalities and assisted more than 1.3 million children and pregnant women from 2016 to 2021 [27]. It aims to improve ECD in the country’s most vulnerable populations by supporting parental practices and increasing the ease of access to other government social services in diverse communities throughout the country. Studies of PCF have shown that it is generally well accepted by mothers and staff, who noted the importance of the program for their children’s development [28,29]. PCF delivers structured home visits by paraprofessional home visitors (HVs) who provide guidance to caregivers on play and responsive care. Municipal teams typically schedule weekly or bi-weekly visits for children 0–3y, adapting cadence to local geography and family needs while using the Care for Child Development guidelines. PCF was developed based on the Care for Child Development, UNICEF model [30]. The Care for Child Development methodology contains broad guidelines for HVs to follow during their home visits. Care for Child Development has been implemented in 54 low- and middle-income countries, with mixed experiences reported about its implementation and ability to deliver with high fidelity and quality [31].

A large study (1,623 children) was conducted in 2019–2020 to assess the implementation and impact of PCF [32]. The study did not find significant improvements in ECD scores and revealed multiple problems in implementation. After one year of participating in the program, 31% of the caregivers indicated they had not received a home visit in the previous month and only 24% of caregivers were able to recall any message that was shared with them during a home visit. Program Coordinators at the State-level indicated that only 30% of the municipalities that were operating PCF had “strong implementation” [32]. Another study was conducted to look more closely at the implementation of PCF in large urban settings. The data collection took place in 2021, while many of the home visits were still being conducted virtually. The study identified barriers related to family’s knowledge of PCF program goals, and lack of referral protocols to connect families to social services [33]. Key findings from the evaluations of PCF that have been conducted can be seen in S1 File.

Findings from large-scale parenting and home-visiting programs show that once initiatives operate at national or statewide scale, persistent implementation barriers often attenuate intended impacts [34–43]. Within the broader literature on scaled parenting programs, it is the post-scale-up implementation phase that is most vulnerable, when fidelity, workforce stability, and service coordination are hardest to maintain [44]. Large-scale home-visitation programs therefore require close monitoring and continuous, data-informed adjustments to sustain high-quality services, particularly when serving diverse populations [45–48]. Most evaluations of PCF were conducted in large cities or state capitals, although PCF predominantly operates in small and rural municipalities, leaving a gap in evidence on barriers limiting impact in these settings. Additionally, most studies were conducted before or during the pandemic period when field activities were suspended, and therefore, there is little knowledge about what barriers still exist or have been newly created following the return to in-person home visits after the COVID-19 pandemic. The primary aim of this study was to identify implementation barriers of PCF in rural and small urban areas that are reported by program participants. The results offer multidimensional insights to inform future program adaptations and the development of targeted implementation strategies. These results provide practical, generalizable guidance for home visitation and analogous early-childhood programs operating in small- and medium-sized, resource-constrained settings worldwide.

## Materials and methods

### Data collection and research participants

This study was developed in partnership with the National Secretariat of Early Childhood (Secretaria Nacional da Primeira Infância) of the Ministry of Development and Social Assistance. Data for the analysis of barriers was gathered through key informant interviews (KIIs) and focus group discussions (FGD). Recruitment began on 01/12/2021 and ended on 02/20/2022 (DD/MM/YYY).

A total of 11 municipalities and three state coordinators participated in the KIIs and FGDs. The municipalities were selected to represent a diverse range of characteristics, including rural and urban, medium and small municipalities, diverse cultural identities, and logistical accessibility from the five regions of Brazil. The research team used purposeful sampling to select participants and assign them to the KII or FGD groups from each municipality to ensure a diversity of perspectives and that all essential positions within the PCF structure were represented. The participants had to satisfy two inclusion criteria: 1) working as a member of PCF or receiving the program service as a beneficiary or 2) having in-depth knowledge of PCF operations, at their respective levels of operations.

All municipalities involved had returned to in-person home visits during the study period. Much of the training was still being conducted virtually, as this was seen as a resource-saving approach to training. For recruitment, municipalities were asked to nominate participants for the interviews that represent key actors in their territory, including beneficiaries, HVs, supervisors, social assistance secretariat, and state-level coordinators. All supervisors from each municipality were interviewed as key informants. At least one beneficiary (caregiver or pregnant woman) was recruited from each municipality to participate as a key informant. All home visitors participated in the focus group meetings. This design ensured representation across all key positions within the PCF delivery structure and across diverse municipal contexts. Participants were recruited until the research team assessed that thematic saturation had been reached (see Results). The invitations to participate in the study occurred through telephone contact, WhatsApp messages, and e-mails. After recruitment was confirmed, the KIIs and FGDs were conducted online via video conferencing software.

The interview guides for the key informant interviews and the focus group discussions were developed by the authors and tested with a small number of participants before conducting the study. The questions in the interview guides were based on the Home Visiting Workforce Needs Assessment Framework (further described in the analysis section below) [49].

All interviews and focus groups were conducted by two Brazilian researchers with experience in qualitative research and knowledge of the PCF using an open-ended interview guide. At the beginning of the session, the interviewers introduced themselves and provided each potential participant with a verbal explanation of the study’s purpose, procedures, risks, benefits, and their rights as a participant. The interview guide provided the text for the interviewer to read, which was approved by the IRB. The study participants were given the opportunity to ask questions and receive answers to ensure informed decision-making. Once all questions were addressed and the participant voluntarily agreed to participate, verbal consent was obtained and documented by the interviewer, including the date and time of consent. Following verbal consent, they began the interview. The project was approved by the national ethics committee of Brazil (CONEP) and the PI’s institutional ethics committee at Hospital das Clínicas da Faculdade de Medicina USP (CAPPESQ - HCFMUSP) and approved under the protocol number CAAE 51767121.1.0000.0068.

### Data analysis and interpretation

The current study used the Framework Method, a qualitative descriptive approach that emphasizes low-inference, data-proximal analysis. The aim was to summarize participants’ experiences and identify actionable implementation barriers, rather than engage in deeper interpretative or theory-building analysis. The study included deductive and thematic content analysis to investigate barriers to the implementation of PCF [50]. The approach is used to draw descriptive and explanatory conclusions clustered around themes. It includes the creation of a matrix output to summarize data, providing a structure for the researchers to systematically synthesize the data by case and by code [51].

The analysis aimed to provide a diagnosis of PCF implementation procedures and identify program challenges, bottlenecks, and potential points of improvement. The codebook for the analysis was developed based on two analytical frameworks: the Home Visiting Workforce Needs Assessment (HVWNA) [49] and the Consolidated Framework for Implementation Research (CFIR) [52]. The two frameworks were selected based on the perceived fit they exhibited between the objective of the study (evaluate implementation determinants) and the nature of the program being evaluated (home visitation program). The HVWNA was created by The Early Childhood Workforce Initiative with the aim of developing a framework that will help identify implementation challenges related to the workforce. It includes seven dimensions of evaluation, (1) expectations of the workforce (2) curriculum content, materials and resources; (3) training, supervision and career development; (4) working conditions; (5) program design; (6) favorable environment; and (7) quality monitoring and assurance. The seven dimensions serve as a guide for the qualitative analysis of PCF and for identifying focal points for structural modifications [49]. The CFIR was used because it provides a a determinant framework that specifies constructs that can influence implementation outcomes across domains (e.g., innovation characteristics, inner setting, outer setting, individuals, and implementation process). It provides a framework for systematically organizing and assessing potential barriers and facilitators. The framework can be used to guide the development of implementation strategies and identify key adaptations that are needed [53]. The CFIR includes 5 domains of analysis:; the innovation, outer setting, inner setting, individuals involved, and the implementation process. Each domain includes constructs that influence implementation. The domains served as categories to organize the barriers that are identified through qualitative analysis.

We developed a coding matrix that incorporated HVWNA’s seven dimensions as one analytic lens for organizing home-visiting–specific barriers [49] and CFIR’s five domains as a complementary lens for organizing determinant levels [52,53]. Coding was conducted at the level of data excerpts (coded transcript segments). This excerpt-level dual coding was used to improve analytic completeness and to reduce the likelihood that barriers specific to home-visiting delivery systems would be missed while also preserving a generalizable determinant structure [49,52,54,55]. Coded excerpts were clustered into barrier themes. For reporting, each barrier theme was assigned one primary HVWNA dimension indicating where the barrier sits within the home-visiting delivery system and one primary CFIR domain indicating the determinant level at which change levers and implementation strategies are typically targeted. Secondary HVWNA/CFIR mappings were noted when barriers consistently spanned multiple dimensions/domains. We report results organized by the seven HVWNA dimensions and provide two complementary summaries: Table 2 (barriers categorized by CFIR domain) and S5 File (an integrated HVWNA–CFIR crosswalk linking each barrier to both frameworks).

**Table 2:** Barriers categorized by CFIR Domains.

**CFIR Domains and Barriers**
**Domain: Innovation Characteristics: Features of the intervention itself (e.g., evidence strength, complexity, cost) that influence implementation.**
Lack of a structured curriculum in the program protocol with suggestions of age-appropriate activities to be performed during the home visit.
Difficulty adapting the home visit content to diverse local contexts and families’ culture.
A need for content and guidelines on the visit’s adaptation for children with disabilities.
The HVs and the families sometimes lack household material/toys to be used for the activities they selected for the visit.
Beneficiaries expressed that they would like more frequent and longer lasting home visits, while HVs expressed that it is difficult to conduct the suggested frequency and duration of home visits due to competing responsibilities and logistical difficulties.
**Domain: Outer Setting: External context and pressures—such as policies, funding, and stakeholder needs—shaping implementation.**
The interns’ contracting modality poses a barrier due to restrictions regarding workload (hours) and contract duration, resulting in high turnover.
Implementing PCF is not mandatory for the municipalities and so adherence depends on political will.
The delay in registering newborns at the national social program can prevent families from receiving visits during a very important period of early childhood development.
**Domain: Inner Setting: The organization’s internal context, including culture, climate, resources, and readiness for change.**
Many families do not have appropriate material/toys in the household to practice the activities selected by the HVs.
The long distances between houses can make it difficult to conduct the home visits with the frequency and duration that the program requires.
The transportation for the home visits is not always provided by the municipality so it can be difficult for HVs to reach the beneficiaries due to limited transportation options.
Program funding does not consider the distance or difficulty to reach some communities and houses. This especially limits the ability to serve traditional communities outside urban areas.
The extreme vulnerability of some families can cause a heavy emotional burden for the HVs, who have limited support from the supervisors or the social unit.
High turnover rates make it difficult for new team members to receive effective training
Lack of a structured supervision process and the fact that supervisors often have other work responsibilities outside of PCF hinders their ability to provide quality services to PCF.
**Domain: Characteristics of Individuals: Knowledge, skills, beliefs, self-efficacy, and other attributes of people involved in implementation.**
HVs may have difficulty delivering effective home visits to families with cultures distinct from their own. Language and cultural barriers can arise when delivering visits to traditional communities.
The extensive cultural diversity of the program beneficiaries makes it difficult to apply the home visit protocol to all families. HVs expressed the need for adaptation of program protocols and materials to accommodate diverse contexts.
**Domain: Process of Implementation: The activities that drive implementation—planning, engaging stakeholders, executing, and reflecting/evaluating.**
The selection process for choosing which families receive PCF is not systematic and often only includes the families that live closest to the municipality center to facilitate visit delivery.
HVs receive the initial training from supervisors that received little training and have no pedagogic training.
The training needs to incorporate more topics from experts and specialist that are important to providing effective home visits and program delivery.
The training agenda and visit guide needs additional information regarding how to provide effective visits for children with disabilities
The monitoring system still contains paper-based instruments which causes difficulties for the implementors.
HVs have difficulty in understanding and administering the child development monitoring instrument.
The staff receives no training on monitoring instruments, including the electronic system. An additional module on monitoring is recommended.

We use implementation to refer to the process of putting an intervention into routine practice and the determinants that influence that process (i.e., factors affecting adoption, delivery, and sustainment). Accordingly, when we use the phrase implementation barriers, we mean barriers to the delivery and sustainment of PCF home visiting as intended, regardless of whether they originate in the intervention itself (innovation characteristics), the inner setting, the outer setting, individuals, or the implementation process. When referring specifically to CFIR’s Process domain, we use the term implementation process.

The interviews were recorded and transcribed by one researcher and proofread by four members of the research team. To establish coding consistency, two researchers trained in qualitative analysis (CMW and ALRC) independently coded the same three KII transcripts and one FGD. Disagreements were discussed systematically until consensus was reached on each code, producing a refined codebook and shared coding conventions that defined how each construct should be applied. This consensus-building process governed all subsequent coding. Following this established procedure — consistent with the Framework Method approach (Gale et al., 2013) — one researcher (CMW) coded the remaining transcripts using the consensus-refined codebook. Upon completion, all coded excerpts were extracted from Atlas.ti and organized into a structured results matrix in Excel, with quotes categorized by theme within each framework dimension. To provide analytic validation across the full dataset, three researchers with expertise in home visitation programs and direct knowledge of PCF (CMW, ALRC, and AVMB) independently reviewed the results matrix, cross-checked thematic interpretations, and collaboratively resolved any discrepancies in categorization or emphasis. The most analytically important excerpts were selected through this team review process. The reporting of findings adheres to the reporting guidelines of the Standards for Reporting Qualitative Research (SRQR) [56].

The study was approved by the national ethics committee of Brazil (CONEP) and the PI’s institutional ethics committee at Hospital das Clínicas da Faculdade de Medicina USP (CAPPESQ - HCFMUSP) and approved under the protocol number CAAE 51767121.1.0000.0068.

## Results

In line with our dual-framework analytic approach, S4 File summarizes and defines each Home Visiting Workforce Needs Assessment (HVWNA) dimension used to organize the Results and identifies the corresponding Consolidated Framework for Implementation Research (CFIR) domain definition used for cross-framework interpretation.

A total of 6 FGDs and 11 KIIs were analyzed, involving 38 participants across the 11 municipalities and including the three state-level coordinators referenced in the Methods. Participation was stopped at this point because the research team determined that they were experiencing thematic saturation [56]. The KIIs lasted between 40–70 minutes and the FGDs lasted between 50–90 minutes. The FGDs included 3–6 participants. The list of municipalities and number of participants in each municipality is presented in Table 1. A total of 38 participants took part in the FGDs and KIIs. The participants included State Coordinator of PCF, Coordinators of Social Programs at Municipality, Social Assistance Secretary, PCF Steering Committee members, Supervisors of PCF, HVs, Pregnant Beneficiaries, and mothers. The participants’ location, role, and years of experience with PCF can be seen in S2 File.

**Table 1:** Participant’s municipality.

Participant’s Municipality	State	Region	Number of Participants
Agua Branca	Alagoas	Northeast	4
Amazonas	Amazonas	North	1
Cidade Ocidental	Goiás	Central-West	3
Felisburgo	Minas Gerais	Southeast	1
Goianapolis	Goiás	Central-West	3
Ilha Comprida	São Paulo	Southeast	6
Jacareacanga	Pará	North	4
Petropolis	Rio de Janeiro	Southeast	9
Roncador	Paraná	South	1
Ribeirão Pires	São Paulo	Southeast	1
Tocantinia	Tocantins	North	5

The results provide a diagnosis of PCF by identifying program challenges, bottlenecks, and potential points of improvement. The results that are most important for understanding the barriers to the program or providing the most potential to lead to program adjustments are presented here. The complete set of quotations that were extracted from the transcripts, along with the original Portuguese text, can be seen in the Results Matrix in S3 File. Results are presented according to the seven HVWNA dimensions [42] to retain a home-visiting–specific, program-improvement orientation. In parallel, each barrier theme was mapped to CFIR domains to describe the determinant level shaping implementation. Table 2 summarizes barriers by CFIR domain, and S5 File provides an integrative crosswalk that links each barrier theme simultaneously to a primary HVWNA dimension and a primary CFIR domain, with common secondary mappings where relevant.

1 Workforce Expectations

The Workforce Expectations category provides the opportunity to reflect on how clearly the program describes the tasks that are expected of HVs, the way in which competencies and standards are used to inform training and professional development, and the process used to recruit and hire qualified candidates. The Social Program Coordinators at the municipal level oversee the implementation of PCF by recruiting short-term workers, assigning existing municipal staff to PCF, contracting interns from universities, and hiring personnel from other local social programs. The hiring process must adhere to national guidelines, which specify contract details and minimum requirements for each position. However, there is room for flexibility to accommodate various contexts. Some study participants highlighted challenges in staffing PCF. For instance, there are no specific capacity requirements for supervisors and so underqualified individuals may be appointed to the position.

A Coordinator of Social Programs stated, *“the issue of hiring makes it very difficult for municipalities, very much. I think it should be more simplified, most management (supervisor) positions are not political positions, but unfortunately sometimes end up in the hands of people who are not prepared at all. And the manager makes a difference because it’s the manager who will fight (for the program), who will request things from the mayor, who will go after everything for the organization. So, I think the hiring process should be much more simplified. They have made it worse now because they are saying that we can no longer hire third parties.”*

2 Curricula, Materials, and Resources

The Curricula, Materials, and Resources category of the HVWNA provides the opportunity for the program to reflect on the accessibility and quality of curricula, materials, and resources that are provided by the program to the HVs and the families they work with. PCF uses the Care for Child Development methodology (UNICEF), which contains general guidelines for home visits and recommends that trainers and HVs follow the protocol while conducting home visits. However, several participants described limitations of the guidelines and difficulties planning activities for the home visits. They mentioned that the program protocol does not provide sufficient information on what activities should be conducted and what materials should be used. There is no curriculum which includes a collection of suggested activities based on the age group of the child or for pregnant women, but only a few examples. The program also does not define a toy kit or set of materials to be used during the visits. It was reported that HVs search online and in books for ideas for activities to conduct during the home visits and prepare materials or toys with available supplies.

One supervisor said, “W*e research on YouTube for some type of activity, and we create the material with what we have on hand and bring it to the next visit. We do everything, research everything”*

Participants mentioned the difficulty of following the Care for Child Development guidelines for home visits because they did not align with the cultural reality of some families. The participants described difficulties selecting activities and using suggested materials to attend to the diverse populations.

One supervisor said, *“It is a very big challenge when we apply the method with families, which often does not align with the reality of that family. It does not correspond to their reality. Thus, I see a lot of difficulty for visitors in relation to this.”*

A supervisor said, *“one difficulty we had with the indigenous people who live here in the city, and there are many, was the language. They don’t speak Portuguese very well. Now, we have a visitor who is bilingual.”*

HVs are encouraged to use materials that the families have in the household to conduct the activities. Several participants described difficulties with obtaining appropriate materials to use during the home visits. It was mentioned that many families do not have appropriate materials in the household and so the HVs must create toys with material from their own homes or propose activities with limited material they find in the beneficiary’s home.

A home visitor said, *“Many times you have to take some materials from [our] home; this is something that I have experienced a lot. I have also gone through situations where I end up taking some things from home because that family doesn’t have those materials to carry out the activity.”*

A supervisor said, *“… we have to make some adjustments because there are activities that are related to the material that the family has in their house. But we visit families that don’t even have a place to sit, where the family doesn’t have any pots to do that activity. So, we have to think about different activities to help explain things to that family.”*

3 Training, Supervision, and Career Development

The Training, Supervision, and Career Development category provides the opportunity to reflect on the accessibility and relevance of existing training and supervision for HVs and supervisors, along with career advancement opportunities. PCF recommends ongoing training for the HVs but allows the State offices and municipalities to determine the details of the training. However, the initial training protocol when onboarding HVs is standardized and mandated, which follows a “cascading training model” in which national-level teams train state-level teams, state-level teams train municipality-level supervisors, and the supervisors train the HVs. Therefore, the highest levels (national and state) receive training from the most qualified individuals, and the lower levels (HVs and supervisors) receive training from less qualified individuals. The quality of training likely diminishes after each level of knowledge transition. Study participants expressed reservations regarding the quality of training that is delivered by the program.

A Coordinator of Social programs said, *“Unfortunately, the issue of the visitors [training] has always been a big problem within the program because of the training for them. There was never face-to-face training, it was always the supervisor, or the manager, or the coordinator who received training and brought [the training] to the municipality.”*

The problem with the quality of training is further complicated due to the high turnover rate of professionals at the municipality level. Therefore, the state and national level trainers have difficulty to properly train new staff. The supervisors, even the ones recently hired, conduct training for the visitors in most of the municipalities, leading to lower quality training. One of the primary reasons for high staff turnover is the use of interns by municipalities. Additionally, the frequent change of political parties in municipalities often leads to new appointments of personnel within the PCF positions.

A State Coordinator said, *“The constant turnover of professionals occurs in the municipalities, where changes in [political] management occur. It wasn’t just the supervisor and visitor who were replaced. In most of our 24 municipalities, the entire team was changed. So, when someone left, they didn’t stay to pass on [the knowledge] to the next person, you know?”*

Study participants mentioned they wanted more information in their training sessions and that they lack some information to effectively conduct their tasks, such as specialized training on topics of early childhood development and how to provide services to children with disabilities.

A Supervisor said, *“We should invite experts [to conduct trainings], perhaps an educator, so that we can create a half-day or a full-day program, because when we search for things, knowledge, and foundational training, for example, social interactionism, cognition, motor skills, visual, fine motor skills. Imagine someone who is not from the field trying to understand these terms, right? You either ask questions, go to the dictionary, or start researching other articles.”*

The training modality that was implemented during the COVID-19 pandemic was primarily virtual (initial 40 hours) and virtual training continued after COVID-19 in some locations to facilitate easier logistics. Some participants expressed that there were benefits to the virtual training modality, primarily high-level administrators. While study participants at the municipality level (HVs and supervisors) primarily expressed difficulties and dislike for the virtual training sessions.

A supervisor said, *“We do the necessary training, but everything is very virtual. So, I felt a bit that there is a difference and difficulty in that; we have to make much more effort to achieve effective learning.”*

4 Workforce Conditions

The Workforce Conditions category gives the program the opportunity to reflect on compensation, mechanisms for recognizing the workforce, workload, and organizational culture in the program. The workload of PCF implementers is limited by policy that dictates that a visitor can attend a maximum of 31 families and a supervisor can supervise a maximum of 15 visitors. However, the number of families that a HV can work with is determined by the municipalities depending on the characteristics and distance of the families. Home visitors are responsible for planning the home visit activities, conducting home visits, participating in team meetings, completing the required documentation, and giving referrals to other services. There’s no single national pay rate for PCF HVs. They’re hired by municipalities, so salaries vary by locality and year. Recent municipal notices show monthly pay typically around R$1,300–R$1,900 a month, with supervisors earning more.

In some municipalities, the supervisor is not full-time dedicated to the program, having other obligations in the social service unit. Several supervisors expressed a limitation in the amount of time they have available for their responsibilities due to working in multiple roles, in multiple capacities. For example, some supervisors work as supervisors for PCF and as managers of other social programs within the social service unit.

A supervisor said, *“I end up doing two things at the same time, right? I have half of my time dedicated to the program [PCF] and half dedicated to the management of the social program registry system [Sistema unico], which is for social assistance. So, sometimes this management part requires more attention, and I can’t dedicate as much time to the program.”*

5 Program Design

The Program Design category reflects key aspects of the program’s design including its target population, intensity of services, and content. A barrier that was expressed by study participants related to the program design is created by the methodology for selecting communities and individuals that receive PCF services. The Program covers vulnerable families enrolled in the national cash transfer Program (Bolsa Familia). According to the municipality’s size and number of potential candidates, the goal of participants is set by the Federal level. Currently, municipalities must opt-in to participate in PCF and they select which communities and individuals within their district receive PCF. Some municipalities only provide PCF services to a small portion of the eligible families in their district. There is no policy in place for a systematic, data-driven process for selecting who receives PCF. For example, study participants noted that communities that are distant from the urban centers of the municipality do not receive PCF.

A supervisor said, “.. we only attend those that live here, we do not have families [in PCF] in the villages because of the long distances.”

The program is offered on a weekly basis for children from 0-3 years and bi-weekly visits for pregnant women and for children with special needs from 0-6 years. However, the frequency and duration of home visits was often debated by the study participants. PCF beneficiaries often expressed that they want to receive more visits and visits of longer duration. HVs often expressed that they prefer less frequent visits (not weekly) and often cannot conduct visits for the full duration of time that is recommended, due to the difficulties of reaching beneficiaries.

A beneficiary said, *“I think it is great to have weekly visits, because I see that my child’s development is weekly.”*

A home visitor said, *“I thought the proposal that the home visit does not occur weekly was great. I thought the proposal was good.”*

A program manager said, *“It [the program] was not planned based on the characteristics of the municipalities, no? Like the topic of 45-minute home visits. What they hoped for when creating the program was that one house was next to the other house, that the visitor was able to attend them. Today, each visitor attends 25 families, that is their goal. That would be 5 families per day. They hoped that those families lived close, I imagine, to allow the necessary time for the visits, but it does not work that way.”*

6 Favorable Environment

The Enabling Environment category reflects on the overall context in which the program operates, including how responsibilities are divided among levels of government, buy-in for the program, available funding, and the capacity of leadership. Eligible municipalities (municipalities with at least 100 vulnerable families and a social service unit) can choose if they want to implement PCF because it is not a mandated social program. Some study participants expressed that the volunteer nature of PCF implementation reduces the reach of the program because it depends on political will.

A Social Program Coordinator stated, *“In my assessment, the difficulty for the municipality to adopt it [PCF] is because it is optional, not an institutionalized thing like [the public social assistance system]. Some think that because [the municipality] has daycare, pre-school, etc., they can incorporate the fundamental aspects of [PCF] in those services and do not need to implement [PCF].”*

The funding mechanism of the program creates barriers to offering PCF to some families, especially traditional community families. The funding protocol allocates resources based on the number of families served and home visits conducted. The protocol does not take into consideration the distance or difficulties to get to the house but instead provides a standard fee that can be charged. This creates incentives to provide the PCF services to families that live close to the units/visitors or live in houses that can be easily accessed. The most distant homes, which are often the most in need, are often overlooked.

A Social Program Coordinator said, *“Considering our territory, I think our resources should be in line with our territory. Because there are municipalities that have the same number of users as we do and receive the same amount of resources that we receive, however, if we analyze where these families are living, it is very disproportionate, because I have families here that live almost 80 kilometers away.”*

A supervisor said, *“There is a significant indigenous population here in the municipality and the majority, let’s say around 80% of the children in the registry here in the municipality are indigenous. So, it would be important, very important I believe, to extend the program to indigenous communities. Currently, it is focused mainly here in the city, right? We serve indigenous people here in the city, not those in the village, due to the logistics of travel and the need for a larger team to work.”*

Study participants expressed a barrier related to providing PCF services to families with a newborn. To receive home visits by PCF the child must be registered in the social program registry (CadUnico). Study participants explained that there is a long delay at the Federal level from registering newborn babies into the system and they cannot be visited (or have the visit registered for reimbursement) until they appear in the system. The delay can result in the family not receiving PCF services for the first three months of the child’s life, even if the mother was attended by the Program during pregnancy.

A Social Program Coordinator said, *“When we register a family into CadUnico [social program registry system] we go there, the center sees that there is a child in the house that has the age for the program [PCF], they send [the information] to us and we register them in CadUnico. It takes 90 days for the system to determine if the child is a part of CadUnico. So that also delays us because we are with the family on standby, ready to assist but we can’t assist fully because, although we provide assistance to some, it does not count as a visit [of PCF].”*

7 Quality Monitoring and Assurance

The Monitoring and Quality Assurance category gives the opportunity to reflect on how comprehensive monitoring and quality assurance systems are upheld in the program, the capacity in which they are implemented, and the way they are used to ensure sustainability of the program. An electronic registry system for PCF was established in 2020 to improve the organization and analysis of incoming data. The State-level coordinators are responsible for monitoring the data and conduct on-site monitoring to verify compliance of the program protocol. Several barriers were described that involve the monitoring and supervision system.

Not all monitoring instruments are electronically based, so the staff were required to conduct monitoring and reporting with a traditional pen and paper method. Some study participants described difficulties with conducting the paper-based monitoring and storing the documents.

A supervisor stated, *“I asked if I could maintain the registry only on the electronic-PCF system, which is the system to record visits. But I was denied. For now, you have to fill out the plans, the sheets, and everything. I said, it’s a lot of paperwork. A lot of paperwork. We also don’t have any more space to store so much paper here at the CRAS [social programs office].”*

Several study participants explained that the monitoring instruments are complicated and challenging for many HVs and they are not covered by the initial training. Some participants indicated that additional training was needed for HVs to better understand how to use the monitoring system and why monitoring and quality assurance are important.

A Social Program Coordinator said, *“Now with the electronic PCF system, I think we’ll have to adjust the training because there will need to be a dedicated day for this system. The system completely reflects the quality of the program in the municipality, whether looking at visits, teams, and especially because it reflects in the financing.”*

Among the monitoring instruments, the visitor needs to evaluate child development periodically, using a specific child development instrument created for the Program. However, the child development assessment form has been challenging for the HVs. The HVs have reported not understanding the utility of the evaluation and found the evaluation forms difficult to understand and redundant.

### Implementation barriers by CFIR Domains

The barriers identified above describe difficulties that the PCF implementors face while providing home visiting service to the families. The barriers influence multiple aspects of the program. Each barrier was categorized by the CFIR domain it most strongly influences in Table 2. A total of 29 barriers were reported with the Process of Implementation domain providing the most barriers (8 barriers) and the Innovation Characteristics domain and Inner Setting domain providing the second most (7 barriers each). The barriers related to innovation characteristics were primarily focused on a lack of quality material to use during the home visits and frequency/duration of home visits. The barriers related to the Outer Setting domain were related to restrictions placed on the program by national policies/regulations of staff hiring. The barriers related to the Inner Setting domain were primarily related to difficulties arriving to the homes to conduct the home visit and working conditions of the PCF staff. The domain of Characteristics of Individuals provided barriers related to cultural diversity of individuals/families in the target population. The barriers related to the Process of Implementation domain were primarily related to training and the monitoring process.

## Discussion

Across 11 municipalities and 6 FGDs/11 KIIs, we identified a set of recurrent implementation barriers affecting PCF delivery. The barriers span multiple determinant levels and home-visiting system components, reflecting that quality implementation in large-scale, paraprofessional home-visiting systems is shaped by both program design features (e.g., curriculum structure and adaptation guidance) and delivery-system constraints (e.g., workforce stability, supervision capacity, territorial access, and monitoring usability). Importantly, several of the most persistent barriers are rooted in structural and contextual factors that extend beyond program design: workforce instability driven by short-term contracting regulations and political appointment cycles; resource constraints shaped by flat funding mechanisms that fail to account for geographic heterogeneity; and governance complexity arising from the voluntary, multi-tiered federal-state-municipal delivery structure. These broader determinants are discussed in depth in the domain-specific sections below and are important for contextualizing why many of the identified barriers have proven difficult to address through program-level adjustments alone. The most consistently reported barriers were: (1) lack of an age-sequenced, activity-rich curriculum and materials; (2) workforce constraints (short-term contracts, turnover, long travel, limited supervision/training); (3) monitoring systems that are paper-based and hard to use for quality improvement; and (4) exclusion of remote families due to travel/time targets. Program adaptations should emphasize strengthening standardized tools and supervisory systems while preserving necessary local adaptation.

Many of the barriers revealed in this evaluation of barriers reflect problems that have been reported from other home visitation programs globally, and from previous studies of PCF. The current study contributes to the body of knowledge by providing a unique analysis of the PCF program following the COVID-19 pandemic lockdowns. Additionally, a key contribution of this study is the explicit integration of two complementary frameworks: HVWNA and CFIR. HVWNA provides a home-visiting–specific, operational lens that localizes barriers within the delivery system [49]. CFIR provides a generalizable determinant lens that indicates the level at which barriers operate [52–55]. Used together, the frameworks support both program improvement (HVWNA: where to intervene in the home-visiting system) and cross-program generalization/strategy selection (CFIR: what determinant level to target and which classes of implementation strategies are likely to be relevant). Importantly, dual coding was used at the excerpt level as an analytic strategy to ensure completeness; it is not intended to imply that programs must attach numerous framework codes to each barrier in routine practice. To make the integration practical and parsimonious, S5 File crosswalks each barrier theme to one primary HVWNA dimension and one primary CFIR domain (with secondary mappings where barriers consistently span multiple domains/dimensions). This crosswalk functions as a decision aid: for any given barrier, the HVWNA mapping indicates which home-visiting system component should be strengthened, while the CFIR mapping indicates the determinant level at which change levers and implementation strategies should be targeted.

Because ‘implementation’ is used inconsistently across literatures, we distinguish between implementation (the broader set of determinants affecting delivery and sustainment) and implementation process (CFIR Process domain). Our use of ‘implementation barriers’ refers to the former. Consistent with current guidance, we interpret CFIR domains using the updated CFIR (2022) definitions (Damschroder et al., 2022), which clarifies boundaries among domains and reduces overlap across constructs [55]. We use CFIR in the Discussion as an organizing lens to interpret implementation determinants identified in our HVWNA-structured Results. The Discussion, based on the findings from this study, along with the literature and expert opinion, uncovers what PCF can do to improve implementation and impact, and what other home visitation programs globally can pilot and test to address many of the reoccurring implementation barriers [57–59].

### Innovation characteristics domains

In our integrated framework alignment (S5 File), barriers mapped to the CFIR Innovation Characteristics domain aligned most directly with the HVWNA Curricula, Materials, and Resources dimension. Consistent with CFIR (2022), we interpret Innovation Characteristics as determinants inherent to the intervention package itself (e.g., content, specificity, complexity, and adaptability guidance), and we distinguish these from determinants of how the intervention is delivered (Inner Setting), the broader policy/administrative context (Outer Setting), and the activities of planning/executing/reflecting (Process). These barriers reflect limited design specificity and high adaptation demands in a program intended for diverse contexts, including insufficient guidance for disability-inclusive home visiting and for culturally/linguistically diverse settings. The PCF program protocol provides general guidelines for HVs, but does not offer a repertoire of evidence-based material and age-appropriate activities to be used. This results in the HVs seeking out material and examples of activities from third-party sources, such as YouTube, which are not reviewed or proven to be age-appropriate nor effective for families. PCF follows the Care for Child Development curriculum to inform what guidance should be shared during home visits [27]. However, a literature review of global home visitation programs found problems with the Care for Child Development curriculum, including difficulties to ensure implementation fidelity and quality, training, and monitoring [31]. While there is no consensus on the ideal package of home visit guidelines, there is agreement that home visit-based ECD programs should focus on caregiver-child interactions to promote positive parenting [59,60].To guarantee quality and homogeneity across Brazil, PCF could use a more structured curriculum to ensure consistency and effectiveness across the program. The curriculum could provide activities that have been proven to be effective in other settings and are suitable for the different age groups in the target population, while also allowing for flexibility when serving communities and families with diverse needs and backgrounds.

Some HVs of PCF feel overburdened by the responsibilities they are given, which is further exacerbated by a feeling of lack of training and limited materials to provide support. The high number of families per HV, the frequency of home visits, the time required for activity planning and documentation, and the travel time between visits reduces substantially the visit quality. The lack of a structured curriculum contributes to visitors spending significant time planning and preparing activities, decreasing the amount of time dedicated to the visits and often offering inappropriate age-related activities. A previous study of PCF implementation (Buccini, et., al., 2024) found that a lack of resources to support travel to the family homes contributed to the participant’s feeling work overload, and ultimately high staff turnover [33]. To address the issue, PCF could include travel time, planning activities, and time to fill-out documentation as part of the HV’s workday. To allow for more travel time, the frequency of visits could be altered from weekly to bi-weekly, which would ensure that visitors have adequate time to provide quality services to the families they are assigned, without impacting the funding considerably. An RCT conducted in Sao Paulo, Brazil showed promising results for improving ECD through biweekly visits with HVs [14]. This approach should be further tested within the context of PCF.

Supervisors also reported feeling overburdened and unable to complete their assigned tasks with an appropriate degree of attention. Findings presented here regarding the burden created from extensive paperwork, and attempting to utilize an electronic filing system that is poorly understood, were also reflected in a study by Buccini et., al., 2024 [33]. Literature reviews of ECD home visitation programs have shown that adding ECD/home visitation responsibilities to established front-line workers may contribute to poorer retention of workers. This finding has also been seen across other global health programs, displaying a trade-off between improved cost-effectiveness from using existing workers for multipurpose roles, and difficulties related to overburdening the workers [61–64].

### Outer setting domain

Barriers mapped to CFIR Outer Setting were concentrated in the HVWNA Enabling Environment and Workforce Expectations/Conditions dimensions. Political will, funding rules, and external administrative systems shaped municipal adherence, territorial reach, and early enrollment (e.g., newborn registration). We distinguish these from internal resource/capacity constraints (Inner Setting) and from implementation activities (Process). These findings indicate that improving coverage and equity may require adjustments to funding and accountability mechanisms that explicitly account for territorial access and the logistics of reaching remote and traditional communities.

High turnover rates is an implementation challenge that has been commonly reported across global health programs and can be greatly influenced by the outer setting [63,65–67]. The results of this study reflected this common finding. In the case of PCF, the problem is exacerbated by federal regulations on contracts for HVs. HVs are often hired on short-term contracts, such as internships or 12-month contracts. Additionally, HVs are often replaced when there is a change of municipality management through elections. Previous studies of PCF found that high staff turnover was a barrier in all municipalities studies, influenced by low salaries, temporary contracts, and limited benefits [33]. The high turnover rates hinder the establishment of trust between the family and the program and diminishes the quality of home visits due to low levels of training and work experience. Studies on the sustainability and scalability of ECD programs across diverse global health contexts displayed the importance of retaining trained frontline staff to ensure quality implementation and service delivery [68,69]. A study of the Saving Brains grant portfolio and other global health implementation research produced recommendations to address poor retention by implementing strategies including over-recruitment, fast-track training, and provision of high-quality training and supervision [59,65]. PCF could address the high turnover rate by offering greater stability in the contracts, opportunities for career growth within the program, and protecting against staff change when new political assignments are made. Additional workforce retention strategies — such as performance recognition mechanisms, non-monetary professional incentives, and structured progression pathways within the social assistance system — could further support staff morale and continuity, subject to municipal and federal resource availability.

### Inner setting domain

Barriers mapped to CFIR Inner Setting were concentrated in the HVWNA Workforce Conditions and Training/Supervision dimensions. Resource constraints, transportation limitations, staffing instability, role strain, and limited supervisory capacity reduced implementation feasibility and weakened support structures for home visitors working with highly vulnerable families. These workforce barriers are consistent with challenges observed in other large-scale community-based service delivery programs and underscore the need for structural supports that strengthen supervision and reduce worker burden [57–59].

Municipalities that serve hard-to-reach communities have more difficulty providing PCF services than more urbanized municipalities. At the time of writing, federal funding provides R$ 75 BRL per family per month, regardless of how difficult it is to reach the homes. In hard-to-reach locations, the funding does not cover the expense of providing the service, so municipalities have little incentive to enroll those families into their program. These families are often the most vulnerable and culturally diverse. A previous study reflects this finding, stating that some municipalities prioritize enrolling families that are closest to the operating stations of the HVs, or reducing the number of home visits for the families that live further [33]. Inequity in spatial access leads to the most vulnerable populations being underserved or excluded.^62^ Addressing the disparity is critical to promoting health equity [70]. PCF could mitigate the problem of inequity in spatial access by establishing a funding system that provides additional funding to municipalities that serve hard-to-reach communities and increase the per-home visit rate for households with difficult access. Given the complexity of adjusting national funding mechanisms, targeted pilot projects in municipalities with significant geographic barriers would provide valuable evidence on the feasibility and cost-effectiveness of alternative funding and delivery models before wider policy adoption.

### Characteristics of individuals domain

Barriers mapped to CFIR Characteristics of Individuals were closely linked to the HVWNA Curricula/Resources and Training/Supervision dimensions, emphasizing that contextual competence (e.g., disability-inclusive delivery; cultural/linguistic adaptation) requires both better guidance (materials and protocols) and strengthened skill-building (training and mentoring). We distinguish these from gaps in the intervention package and guidance (Innovation Characteristics), organizational supports and resources (Inner Setting), and the implementation activities that build or reinforce capacity (Process).

Program guidelines and material for traditional communities are limited. By leaving municipality and state teams in charge of adapting home visit material for traditional communities, PCF cannot guarantee a quality standard or adaptation procedure for the content. The degree and quality of the adaptation depend on the capacity of each municipality, creating a broad range of results. Adaptations are a necessary part of implementing large-scale programs to achieve a good fit between the evidence-based intervention and the local context. However, adaptation requires a careful and systematic process to ensure the core-components of the intervention remain intact [71]. To address the need, the state and federal teams should contribute to the adaption of home visit material for traditional communities. Additionally, hiring HVs from within the traditional communities can provide a solution to the current language and cultural barriers.

### Process of implementation

Barriers mapped to CFIR Process were concentrated in the HVWNA Training/Supervision, Program Design, and Quality Monitoring and Assurance dimensions. We use the Process domain specifically for the activities by which implementation is planned, executed, monitored, and adapted, and we distinguish these from the intervention’s inherent design features (Innovation Characteristics) and from baseline organizational capacity constraints that shape feasibility (Inner Setting). Cascade training models, limited pedagogic expertise among trainers, and insufficient monitoring training reduced fidelity and constrained continuous quality improvement. Strengthening these processes—through expert-supported training, structured supervision, and simplified monitoring systems—may improve execution and accelerate learning cycles.

The training process for PCF is structured as a cascading model where federal-level experts train the state-level staff who then train the supervisors who train the visitors. The trainers at the municipality level usually do not have experience in child development, pedagogy, or extensive knowledge of the program. A previous study of PCF found that supervisors were uncertain about their ability to provide quality training and replicate the training they received [33]. The training that supervisors and HVs receive ranges greatly in quality and there is no guarantee to the extent the content is covered. The heterogenous and low-quality training at the bottom level creates several difficulties for providing consistent, quality program delivery, and contributes to issues like turnover, low-fidelity and quality of home visits, and ability to supervise and monitor progress. Providing comprehensive training, offering targeted and individualized coaching, and providing reflective and consistent supervision are important components of quality that lead to successfully engaging and retaining families and providing high-quality services [72]. Blended learning approaches that combine expert-led instruction with practical case discussions and structured peer mentoring represent one feasible model for reinforcing core competencies at the front line and should be considered as a candidate for piloting within the PCF training structure.

Assessments of fidelity of home visits and feedback on performance are nearly non-existent in PCF. The supervisors had no standardized protocol or materials to monitor, assess, and report on implementation. Ongoing supportive supervision along with performance feedback have been shown to be critical to intervention quality and fidelity in global health programs worldwide [62,66,73]. For example, when quality enhancement strategies were applied to home visits in a ECD program in rural Colombia, improvements in child development and caregiver practices were made [74]. The observational measure of quality developed by the program has the potential to improve home visit quality at scale [75]. A study of PCF in 2019 reflected similar recommendations, PCF needs to invest in qualified technical support to ensure the quality and fidelity of implementation [76]. PCF could greatly benefit from creating standardized protocols and quantitative instruments to assess implementation and home visit quality. To maximize the effectiveness of any revised monitoring system, training on monitoring instruments — including the electronic platform — should be incorporated as a mandatory component of staff onboarding and included in refresher training cycles, rather than treated as supplementary. The protocol could include obtaining feedback from families, HVs and supervisors. The measures should be reported to the monitoring team at the state- and federal-level, providing information for fast learning and improvement cycles.

### Limitations

The current study faced limitations due to interviews being conducted virtually. The requirements of participating in a virtual interview (computer or mobile device and reliable internet connection) may have constrained availability and contributed to underrepresentation of some stakeholder perspectives, particularly in settings with limited connectivity. Beyond access, the virtual modality may have affected the depth and candor of responses, as the absence of in-person rapport-building can inhibit disclosure on sensitive topics such as staff dissatisfaction or governance failures. Additionally, conducting interviews remotely precluded naturalistic observation of program operations and physical context — information that can enrich qualitative interpretation in implementation research. Future studies would benefit from in-person data collection where feasible, or from supplementing virtual interviews with observational or ethnographic components. Although the study was designed to capture diverse stakeholders across the country, logistical constraints may have limited participation in some contexts. Additionally, selection bias may have been introduced by recruitment for FGDs and KIIs via municipal teams, who prioritized individuals with substantial program experience; this approach may have excluded less-engaged participants, those with dissenting views, or staff with shorter tenure. Combined with the virtual interview modality — which required a device and reliable connectivity — these factors may have further limited representation of the least-resourced implementers and constrained the transferability of findings to settings with weaker institutional capacity or lower program engagement. Future studies using in-person recruitment and snowball or maximum-variation sampling strategies may capture a broader range of perspectives. Additionally, the descriptive nature of the Framework Method analysis means that the current findings identify and categorize barriers but do not support inferences about how barriers interact across implementation levels or which determinants are most influential in shaping outcomes; future research using comparative case designs, realist synthesis, or mixed-methods approaches would be better positioned to address these questions.

With respect to the analytic framework, CFIR provided a useful structure for organizing multilevel determinants; however, systematic reviews have noted limitations, including overlapping constructs, limited attention to workforce-specific determinants, and challenges applying CFIR in large and complex delivery systems (Means, et al., 2020 [54]. These limitations were salient in our context. CFIR’s high-level domains (e.g., Inner vs Outer Setting) can be too broadly specified to cleanly characterize determinants in a national home-visitation system operating across heterogeneous Brazilian municipal contexts, where “setting” spans multiple administrative layers and where territorial access and contracting modalities are central implementation constraints. Prior applications of CFIR can also yield construct overlap if older definitions are applied. In response to this concern, we revised our interpretation of CFIR domains to align with the updated CFIR (2022) definitions (Damschroder et al., 2022), which more clearly delineate domain boundaries and support more consistent domain assignment [55]. Even with these refinements, some determinants remain plausibly interpretable across domains; for example, supervision structures, training cascades, monitoring routines, and turnover-related disruptions can reflect both organizational capacity and climate (Inner Setting) and the execution and learning-loop functions of implementation (Process). This reflects an ongoing challenge in applying determinant frameworks in complex delivery systems, where some determinants are not strictly mutually exclusive and coding decisions require judgement about the primary locus of influence.

Similarly, CFIR domain coverage was uneven in our dataset: barriers clustered predominantly in Process, Innovation Characteristics, and Inner Setting, while fewer mapped to Characteristics of Individuals and Outer Setting, limiting the discriminant usefulness of some domains for summarizing findings (Table 2). To increase conceptual clarity and practical utility, we created HVWNA–CFIR alignment so that each barrier theme is assigned one primary HVWNA construct and one primary CFIR domain (S5 File), and we summarize the HVWNA–CFIR construct alignment in S4 File. CFIR is best interpreted here as an organizing lens for multilevel determinants rather than as a definitive classification system, and its use is strengthened by pairing it with the HVWNA framework to capture home-visiting–specific and workforce/delivery-system determinants that CFIR may specify less directly in this context.

## Conclusions

Quality implementation of PCF is critical if the program hopes to create the improvements in ECD that it sets out to accomplish. The current study provides a multi-dimensional, multi-disciplinary diagnosis of PCF implementation following re-opening from the COVID-19 pandemic in a range of municipality-types. Several implementation barriers were uncovered, but fortunately many of them can be addressed by applying evidence-based adaptations and program adjustments. Recommendations for implementation strategies presented here, and in previous studies, which can address the barriers should be piloted in small-scale implementation feasibility trials before distributed broadly.

One of PCF’s strengths is its ability to adapt to the broad diversity of cultures represented in its population. The program allows for a great deal of flexibility by the municipalities to determine how to best service their distinct populations. The flexibility of the program is also one of its greatest weaknesses. This flexibility results in few standardized procedures, guidelines, or material to support service delivery. The task that the HVs and supervisors are given is complex and sensitive, demanding professional education and training. They do not have sufficient training and support to come up with effective, evidence-based processes, activities, and material to serve the diverse needs of their families. Our results indicate that some elements of program delivery should remain flexible (e.g., adjusting the number of visits based on travel distance), while others should be standardized. Meta-analyses have shown that more standardized protocols and planning guides for ECD programs can help mitigate some of the barriers to deliver quality services [59].

PCF is also limited by policies that are universal to the program and do not account for differences between territories. Funding mechanisms do not consider the distinct challenges that some municipalities face, such as hard-to-reach families and expensive travel costs. The program must find the right balance between adaptability at the local level and providing standardized processes for program delivery that ensures quality and consistency across the entire country.

## Supporting information

S1 File Key findings from interviews. (DOCX)

S2 File Interview participants table. (DOCX)

S3 File Results matrix. (PDF)

S4 File Corresponding domains and definitions. (DOCX)

S5 File Integrated HVWNA-CFIR crosswalk table. (DOCX)
