# Supplementary material for: Implementation barriers of Brazil’s national home visitation program for early childhood development: A qualitative evaluation
Source: PLOS Glob Public Health. 2026 Apr 10;6(4):e0005203. doi: 10.1371/journal.pgph.0005203 (PMC13068277; doi:10.1371/journal.pgph.0005203)
Supplement: S1 File — (DOCX) [file pgph.0005203.s001.docx]

**Supplemental File 1: Key findings from evaluations of Programa Criança Feliz**

| Author | Article Title | Key Findings |
| --- | --- | --- |
| Buccini, et., al., (2024)^1^ | Scaling up a home-visiting program for child development in Brazil: a comparative case studies analysis | Benefits:  -Acceptability of the home visits by participating families  -Perceived improvement in parenting skills.  Barriers:  -Families’ limited knowledge and trust in PCF goals  -Lack of referral protocols to address social needs  -Low salaries, temporary contracts, high turnover, infrequent supervision, lack of an effective monitoring system  -Absence of institutionalized funding was a challenge for sustainability |
| MDS, 2023^2^ | Avaliação qualitativa da percepção de resultados do Programa Criança Feliz junto a seus beneficiários e agentes: Análise de política pública de Atenção à Primeira Infância.  (Qualitative assessment of the perception of results of the Programa Criança Feliz among its beneficiaries and staff: Analysis of public policy for Early Childhood Care.) | Benefits: -Beneficiaries expressed satisfaction with the availability, condition, and usefulness of play and education material  -Beneficiaries are satisfied with the adherence to schedules and the duration of visits  -Beneficiaries have good knowledge of visit purposes and appreciate explanations and referrals to community services.  -Beneficiaries feel supported and valued by the visitors.  -Beneficiary mothers and pregnant women are satisfied with the outcomes of the PCF, perceiving significant changes in child development.  - Pregnant participants reported increased knowledge about healthy pregnancy, greater confidence in childbirth and baby care, and increased paternal involvement.  Barriers:  -The salaries of visitation staff  -The need for more informational materials  -Additional play material  -Consistency in visitation personnel to maintain strong connections  -More frequent home visits |
| Ina Santos, et. al., 2022^3^ | Evaluation of the Happy Child Program: a randomized study  in 30 Brazilian municipalities | The study was interrupted by COVID-19 causing visits to be conducted virtually during the final 12 months of the three-year study  Benefits:  -After year one, ASQ3 scores were not significantly different between groups (0.05 SD higher in the intervention group).  -With propensity score matching (comparing those who had been visited in the past month with those who had not) the intervention group had significantly higher ASQ3 scores (p=0.043)  -After year three, there was no significant differences in ASQ3 between groups (intent-to-treat or propensity score matching).  Barriers:  -After year one, only 31% of children in the intervention group had been visited in the past month.  -Only 24% of mothers were able to recall any piece of advice provided by visitors  -A knowledge test for HVs resulted in a mean score of 5.9 out of 10, and a satisfaction survey resulting in a mean score of 5.3 out of 10  -program fidelity was assessed by evaluating the quality of the home visits. On average, only 9.8% of the listed activities were completed. |
| Gabriela Buccini, et. at., 2021^4^ | Scaling up of Brazil's Criança Feliz early childhood development program: an implementation science analysis | Benefits:  - Changes in the enrollment criteria for families facilitated greater reach for the program  - WhatsApp facilitated networking, capacity building, and adoption  Barriers:  -Operationalizing the intersectoral actions remains a barrier for effectiveness,  -Rushed scale-up generated barriers for quality delivery and led to varying implementation processes across municipalities.  -Legal institutionalization is critical to assure permanent funding and maintenance. |

References

1. Buccini G, Gubert MB, Palmeira P de A, et al. Scaling up a home-visiting program for child development in Brazil: a comparative case studies analysis. *Lancet Reg Health – Am*. 2024;29. doi:10.1016/j.lana.2023.100665

2. Alessandra A, Kocourek S, Battistella LF, Gaviraghi FJ. Avaliação Qualitativa do Programa Criança Feliz: efetividade ou desperdício de investimento? *Soc Em Debate*. 2023;5(1). Accessed June 29, 2023. https://sociedadeemdebate.com.br/index.php/sd/article/view/74

3. Santos IS, Munhoz TN, Barcelos RS, et al. Evaluation of the Happy Child Program: a randomized study in 30 Brazilian municipalities. *Cienc Saude Coletiva*. 2022;27(12):4341-4363. doi:10.1590/1413-812320222712.13472022

4. Buccini G, Venancio SI, Pérez-Escamilla R. Scaling up of Brazil’s Criança Feliz early childhood development program: an implementation science analysis. *Ann N Y Acad Sci*. 2021;1497(1):57-73. doi:10.1111/nyas.14589
