# Supplementary material for: Implementation barriers of Brazil’s national home visitation program for early childhood development: A qualitative evaluation
Source: PLOS Glob Public Health. 2026 Apr 10;6(4):e0005203. doi: 10.1371/journal.pgph.0005203 (PMC13068277; doi:10.1371/journal.pgph.0005203)
Supplement: S2 File — (DOCX) [file pgph.0005203.s002.docx]

Supplement 2. Interview participants location and years of experience

| Transcript Code | Participant’s position in PCF | Participant’s City | Participant’s State | Years with PCF |
| --- | --- | --- | --- | --- |
| FGD 1 | Coordinator of Social Programs | Ilha Comprida | SP | 12 |
|  | Coordinator of Social Programs | Agua Branca | AL | 2 |
|  | Coordinator of Social Programs | Tocantinia | TO | .5 |
| FGD 2 | Coordinator of Social Programs | Ilha Comprida | SP | 5 |
|  | Coordinator of Social Programs | Tocantinia | TO | 2.11 |
| FGD 3 | Home Visitor | Agua Branca | AL | 2 |
|  | Home Visitor | Ilha Comprida | SP | 1.5 |
|  | Home Visitor | Cidade Ocidental | GO | 1.2 |
|  | Home Visitor | Jacareacanga | PA | .25 |
|  | Home Visitor | Jacareacanga | PA | 3 |
|  | Home Visitor | Tocantinia | TO | 3 |
|  | Home Visitor | Goianapolis | GO | 1 |
| FGD 4 | Home Visitor | Petropolis | RJ | 1.8 |
|  | Home Visitor | Petropolis | RJ | 1.2 |
|  | Home Visitor | Petropolis | RJ | .5 |
|  | Home Visitor | Petropolis | RJ | 1.7 |
|  | Home Visitor | Petropolis | RJ | 3 |
|  | Home Visitor | Petropolis | RJ | 2 |
| FGD 5 | Supervisor | Ilha Comprida | SP | 3 |
|  | Supervisor | Agua Branca | AL | 3 |
|  | Supervisor | Jacareacanda | Para | .5 |
|  | Supervisor | Tocantinia | TO | 2 |
|  | Coordinator of Social Programs | Goianapolis | GO | .5 |
|  | Supervisor | Goianapolis | GO | 4 |
|  | Supervisor | Cidade Ocidental | GO | 2.10 |
| FGD 6 | Supervisor | Petropolis | RJ | .11 |
|  | Supervisor | Petropolis | RJ | .2 |
| KII 1 | Steering Committee member | Ilha Comprida | SP | 1.2 |
| KII 2 | Steering Committee member | Ilha Comprida | SP | 1.2 |
| KII 3 | State Coordinator of PCF | Amazonas | AZ | .7 |
| KII 4 | State Coordinator of PCF | SP | SP | 3 |
| KII 5 | Social Assistance Secretary | Felisburgo | MG | .5 |
| KII 6 | Pregnant Beneficiary | Agua Branca | AL | 6.4 |
| KII 7 | Beneficiary | Tocantinia | TO | 1 |
| KII 8 | Beneficiary | Cidade Ocidental | GO | .2 |
| KII 9 | Beneficiary | Petropolis | RJ | 2 |
| KII 10 | Supervisor | Roncador | Parana | 1 |
| KII 11 | Supervisor | Jacareacanda | Para | .6 |
