# Supplementary material for: Implementation barriers of Brazil’s national home visitation program for early childhood development: A qualitative evaluation
Source: PLOS Glob Public Health. 2026 Apr 10;6(4):e0005203. doi: 10.1371/journal.pgph.0005203 (PMC13068277; doi:10.1371/journal.pgph.0005203)
Supplement: S3 File — (PDF) [file pgph.0005203.s003.pdf]

Curriculum Content	English Translation
eu busco alguns materiais que estão disponíveis pela internet, existem hoje muitos blogs, pelo próprio YouTube, muitas pessoas que dão dicas de atividades, o próprio Pinterest, que no início ajudou bastante para eu, que não tinha tanta experiência em confeccionar atividades, então esses recursos acabam ajudando bastante.Hoje a gente tem uma vasta gama de materiais que a gente consegue estar utilizando dentro do Cras, que são vários tipos de materiais, papel, cartolina, enfim, se eu for colocar aqui eu vou ficar o dia todo, mas são muitos materiais, e fora esses materiais que a gente usa para desenvolver essas atividades, a gente tenta também fazer um trabalho com materiais que as famílias têm em casa.	1 I look for some materials that are available on the internet, there are many blogs today, on YouTube itself, many people giving tips for activities, Pinterest itself, which initially helped me a lot since I didn't have much experience in making activities, so these resources end up helping a lot. Today we have a wide range of materials that we can use within Cras, there are several types of materials, paper, cardboard, anyway, if I start listing here I'll be all day, but there are many materials, and besides these materials that we use to develop these activities, we also try to work with materials that families have at home.
A gente usa esse material, o CDC e o GVD, a gente fica o tempo todo com ele, cada visitador tem o seu, eu fico no na minha mesa, porém, para a gente aplicar as atividades, para assumi-la, a gente utiliza sim a internet, a gente pesquisa, o que eu vou fazer com aquela caixa de papelão?	2 We use this material, the CDC and the GVD, we have it with us all the time, each visitor has theirs, I have it on my desk, but to apply the activities, to take charge of them, we do use the internet, we research, what am I going to do with that cardboard box?
Os visitadores, eles utilizam o material para construir as atividades, eles utilizam outros materiais além desses materiais?GESTORA ANA PAULA: Sim, eles sempre estão, como a Núbia é pedagoga, então, assim, ela sempre orienta eles a estarem pesquisando, eles pesquisam bastante na internet, né, como tem a visitadora indígena também, ela passa também bastante algumas experiências dela em relação à cultura indígena, então tem esse diálogo também, tem essa pesquisa por outras fontes também.	3 The visitors, they use the material to build the activities, do they use other materials besides these materials? MANAGER ANA PAULA: Yes, they are always, as Núbia is a pedagogue, so, she always advises them to research, they research a lot on the internet, you know, as there is also the indigenous visitor, she also shares a lot of her experiences regarding indigenous culture, so there is this dialogue too, there is this research from other sources as well.
Sim a gente quando, quando não acha assim o material escrito. A gente vai no YouTube aí pesquisa e procura fazer com o material que tem que tem disponível no momento.	4 Yes, when we don't find the written material like that. We go on YouTube and search and try to do it with the material that is available at the moment.
O material de dados que a gente constrói por conta própria, a gente pesquisa no YouTube, algum tipo de atividade, e com o material que a gente tem em mão, a gente constrói e na próxima visita a gente já leva. A gente faz tudo, pesquisa tudo.	5 The data material we build on our own, we research on YouTube, some type of activity, and with the material we have on hand, we build it and on the next visit we already take it. We do everything, research everything.
E, assim, é um desafio muito grande quando a gente vai aplicar o método CDC nas famílias, que muitas vezes não condiz com a realidade daquela família. Não condiz com aquela realidade. E, assim, eu vejo muita angústia das visitadoras em relação a isso. De acordo também com a questão dos materiais, que, muitas vezes, a gente pensa em algo, mas chega lá naquele momento, não está propício para aquela visita. E, assim, é uma questão muito delicada, que a gente tem que ficar flexibilizando o tempo todo, sem fugir da metodologia do programa	6 And, so, it is a very big challenge when we are going to apply the CDC method to families, which often does not match the reality of that family. It does not match that reality. And, so, I see a lot of anguish from the visitors about that. According to the issue of materials too, which, many times, we think of something, but when we get there at that moment, it's not suitable for that visit. And, so, it's a very delicate issue, that we have to keep flexibilizing all the time, without deviating from the program's methodology.
Como a gente tem 300 e poucas, quase 400 famílias, então, assim, são várias crianças, vários contextos diferentes, gostos diferentes, então, concentrar isso tudo em algumas atividades é bem complicado. E aí a gente está com esse desafio aí para fazer com que seja interessante para todo mundo, e todo mundo realize as atividades propostas para que haja desenvolvimento, que é o foco, né?	7 As we have 300 and some, almost 400 families, so, there are several children, several different contexts, different tastes, so, concentrating all of this into a few activities is quite complicated. So we have this challenge to make it interesting for everyone, and everyone does the proposed activities so that there is development, which is the focus, right?
e ali as meninas já fazem uma observação da criança, qual é o seu desenvolvimento já, para que elas possam buscar as atividades que vão encontrar ali a necessidade da criança. Então, a internet para nós é a principal ferramenta para a elaboração das atividades. Mas tem um grupo aqui do Paraná, que a coordenação estadual criou, que é os supervisores. Eles mandam muita atividade, o que eles confeccionam	8 and there the girls already make an observation of the child, what is their development already, so that they can seek the activities that will meet the child's needs. So, the internet for us is the main tool for the elaboration of activities. But there is a group here in Paraná, which the state coordination created, which is the supervisors. They send a lot of activities, what they make.
Quais atividades têm o mesmo objetivo que de repente na hora pode ser de mais fácil acesso para a mãe? Então, eu tento estar sempre lendo sobre tanto desenvolvimento infantil quanto atividades que estimulem esse desenvolvimento. Os livros para mim são sempre complementares porque, como eu tinha falado, realmente há essa falta dessas informações do marco no programa. Nessas capacitações do programa.	9 What activities have the same objective that might be more easily accessible to the mother? So, I always try to read about both child development and activities that stimulate this development. Books for me are always complementary because, as I mentioned, there really is this lack of information from the program's framework. In these program trainings.
CAROL - E tem algum critério, Eliane, de faixa etária? Existe algum critério que a supervisora estabelece? Pode falar um pouquinho mais?ELIANE - Tem, geralmente colocam as crianças menores, menorzinhas até mais ou menos 6 meses, 7 meses, e as outras atividades, parte de 8 meses até 2, 3 anos.	10 CAROL - And is there any criterion, Eliane, based on age? Is there any criterion that the supervisor establishes? Can you talk a little more? ELIANE - Yes, generally they place younger children, little ones up to about 6 months, 7 months, and the other activities, from 8 months to 2, 3 years old.
ai não eu estou achando que esse conteúdo aqui não é suficiente eu vou aqui, por exemplo, na internet procurar alguma atividade de mãe?<br>SUPERVISORA DÉBORA: Ah, entendi agora. Não, nem tudo a gente encontra lá no manual, certo? A gente tem que levar em consideração a nossa região, nossa realidade daqui, e aí então certas coisas a gente procura pesquisar, procurar outra metodologia para estar trabalhando com a sua família.	11 "Oh, I'm thinking that this content here is not enough, should I, for example, go on the internet to look for some activity for mom? SUPERVISOR DÉBORA: Oh, I see now. Not everything we find in the manual, right? We have to take into account our region, our reality here, so certain things we try to research, look for another methodology to work with your family."
Aqui no meu município, como os brinquedos indígenas é completamente diferente dos não indígenas, a gente vai, as meninas vão para o mato, inclusive a visitadora indígena, né, e ela mesmo que confecciona alguns brinquedos. Outros são confeccionados no momento da visita mesmo com o cuidador e a criança ali do lado. Porque para nós, uma boneca, um carrinho, é comprado, né, e para eles não. Para eles é tudo mesmo justamente para não perder a cultura deles, aquele contato, aquela aproximação com a cultura deles. E aí, então, os brinquedos indígenas são mesmo eles que fazem.	12 Here in my municipality, as indigenous toys are completely different from non-indigenous ones, we go, the girls go into the woods, including the indigenous visitor, right, and she herself makes some toys. Others are made at the time of the visit itself with the caregiver and the child right there. Because for us, a doll, a car, is bought, right, and for them it's not. For them, everything is made precisely not to lose their culture, that contact, that approach with their culture. So, the indigenous toys are made by them.
você tá resgatando a nossa cultura, por que resgatando? Porque eu não levo mais o brinquedo que é da cidade, eu tô produzindo o brinquedo que eu cresci brincando, aquele brinquedo que nós brincava e tudo, então, tudo isso aí eu tô resgatando e eu deixo lá pras mães, pros pais, pra poder eles fazerem, produzirem, né, pra poder as crianças crescerem vendo aquilo ali que o nosso povo fazia, não comprar, porque não tinha dinheiro pra poder comprar o brinquedo, então, o brinquedo já veio.	13 you are rescuing our culture, why rescuing? Because I no longer bring the toy that is from the city, I'm producing the toy that I grew up playing with, that toy that we used to play with and all, so all of that I'm rescuing and I leave it there for the mothers, for the fathers, so that they can make, produce, right, so that the children grow up seeing that there that our people used to do, not buy, because there was no money to buy the toy, so, the toy already came.
Eu acho que as brincadeiras voltadas a alguma, por exemplo, alguma coisa que a gente tenha na nossa cultura, pode ter dança, pintura, corrida de tora, que poderia no caso ser representado por fazer uma tora de isopor, no caso, fazer a corrida, porque a corrida tem um .. que ganha, né? Então brincadeiras relacionadas a isso, acho que seria muito interessante, pinturas, essas coisas. Aquelas brincadeiras de pesca.	14 I think that games focused on something, for example, something that we have in our culture, can have dance, painting, log race, which could in this case be represented by making a styrofoam log, in this case, doing the race, because the race has a winner, right? So games related to this, I think would be very interesting, paintings, those things. Those fishing games.
E essa dificuldade em pensar na atividade, porque eles têm algumas atividades tradicionais. Então, ela passou a conhecer aquela atividade, aquela brincadeira que era comum para aquela determinada região, para aquele determinado povo. Então, foram mais esses desafios que ela colocou. A entrada, a chegada da equipe até essa comunidade, para estar semanalmente na casa daquela família.	15 And this difficulty in thinking about the activity, because they have some traditional activities. So, she started to know that activity, that game that was common for that particular region, for that particular people. So, those were more the challenges she put. The entry, the arrival of the team to this community, to be weekly at the home of that family.
E o bom do projeto é que eles trazem a atividade conforme a dificuldade da criança. Então, tem dificuldade com coordenação motora, só vai trazer coordenação motora, traz outros tipos, mas o foco principal é coordenação motora.Tem dificuldade com a fala, vai trazer os da fala, então assim, ajudou muito por conta disso	16 And the good thing about the project is that they bring the activity according to the child's difficulty. So, if there is difficulty with motor coordination, they will only bring motor coordination, they bring other types, but the main focus is motor coordination. If there is difficulty with speech, they will bring speech activities, so, it helped a lot because of that.
ela não leva brinquedo não, aí eu faço em casa; ele brinca muito com chaves, coisas que faz barulho; ele tem uma bola de plástico que ela sai rolando e vai correndo atrás dela	17 She doesn't bring any toys, so I make them at home; he plays a lot with keys, things that make noise; he has a plastic ball that rolls and he runs after it.
as famílias estão em situações muito mais, assim, a situação de vulnerabilidade aumentou bastante, né, então, eu penso que a gente já tem, já que o programa já deu esse passo à frente de ter, de estar sempre inovando em relação às capacitações, da gente pensar agora em materiais que também estejam conectados com esse período que nós estamos vivendo e também o pós-pandemia	18 Families are in much more, well, the situation of vulnerability has increased a lot, right, so, I think that since the program has already taken this step forward of always innovating in relation to training, we should now think about materials that are also connected with this period that we are living in and also post-pandemic.
o programa Criança Feliz hoje não tenha uma cartilha específica, tipo, se a criança tem síndrome de Down, a atividade vai ser isso, isso e isso. Tem atraso intelectual, a atividade é essa. Acho que hoje a gente não tem esse material. Então, por isso que às vezes a gente se perde nessa parte, adaptou a atividade, mas a gente não sabe o que fazer para passar.	19 Today, the Happy Child program does not have a specific booklet, like, if the child has Down syndrome, the activity will be this, this and this. If there is intellectual delay, the activity is this. I don't think we have this material today. So, that's why sometimes we get lost in this part, adapting the activity, but we don't know what to do to pass it on.
eles sentem muita falta de materiais, inclusive o ministério ficou nos devendo uma cartilha para trabalhar esse público de criança com deficiência para trabalhar esse público de criança com deficiência porque é isso, a gente sempre fala a essência do programa é o vínculo socio efetivo a essência do programa é o vínculo socio efetivo então a gente sempre fala para eles irem nessa linha por exemplo, a gente já se deparou com situações que por falta de informação, por exemplo, da mãe ela não fica muito perto da criança ela	20 they miss materials a lot, including the ministry owed us a booklet to work with this audience of children with disabilities because that's it, we always talk about the essence of the program is effective socio-emotional bonding. So we always tell them to go in that direction, for example, we have encountered situations where due to lack of information, for example, from the mother, she doesn't stay very close to the child.
Eu acho que sim. Até porque assim, aqui na cidade existe muitos indígenas e, eu sou mestiça, sabe? A minha mãe é indígena, então eu não tenho tanta dificuldade em compreender, né? A língua materna. Mas conheço vários indígenas que reside aqui na cidade também, inclusive alguns que participa também desse programa, o programa criança feliz, que muitas vezes fala: "Ah eu não entendo, não sei como é que é, eu não sei o que significa". Então eu acho que botasse um pouco mais, ajudar até mais pra língua portuguesa que é o que as crianças tem muita dificuldade por falar somente a língua … , que é a língua indígena diferente daqui, eu acho que seria interessante	21 I think so. Because here in the city, there are many indigenous people and, I am mixed-race, you know? My mother is indigenous, so I don't have much difficulty in understanding, right? The native language. But I know several indigenous people who also reside here in the city, including some who also participate in this program, the Happy Child program, who often say: "Oh, I don't understand, I don't know what it's like, I don't know what it means". So I think adding a little more, helping even more with the Portuguese language, which is what children have a lot of difficulty with because they only speak the indigenous language, which is different from here, I think it would be interesting
No entanto a gente tem até uma dificuldade na questão dos indígenas também que moram aqui na cidade do que a gente só atende os que moram aqui, a gente não tem famílias na aldeia pela conta da distância muito grande, e aí é uma dificuldade que a gente tinha com os indígenas que moram aqui na cidade que são muitos era na língua né, não falam muito bem português, e aí agora a gente tem uma visitadora que ela é bilíngue.	22 However, we also have a difficulty with the indigenous people who live here in the city than we only assist those who live here, we don't have families in the village because of the very great distance, and that is a difficulty that we had with the indigenous people who live here in the city, which is mostly about the language, they don't speak Portuguese very well, and now we have a visitor who is bilingual.
Aí, pedi um copo, ela não tinha um copo. A gente ficava de coração partido, assim. Aí, um depósito, uma bacia de tampa, né? Para a criança usar a psicomotricidade, o cognitivo. A coordenação motora fina para tampar. Ela não tinha nada Aí.	23 So, I asked for a cup, she didn't have a cup. It broke our hearts, like that. So, a container, a bowl with a lid, right? For the child to use psychomotor skills, cognitive skills. Fine motor coordination to cover it. She had nothing.

Muitas vezes tem que levar alguns materiais de casa isso é uma coisa que eu passei muito, eu passei muito também por isso, de acabar levando algumas coisas de casa porque aquela família não tinha esses materiais para reproduzir a atividade, isso também é um dos grandes desafios que eu percebo que é identificar. Quais materiais aquela família possui, que ela pode reproduzir essa atividade	24 Often you have to bring some materials from home, this is something that I experienced a lot, I also went through this a lot, of ending up taking some things from home because that family didn't have these materials to reproduce the activity, this is also one of the great challenges that I notice which is identifying. What materials does that family have, which they can use to reproduce this activity?
as famílias, elas costumam aderir a essa estratégia, ou é difícil que eles se disponibilizem a construir junto com vocês?GENILDA - Assim, na maioria das vezes sim, muitos não constroem por um motivo assim, não ter materiais como tesouras, porque às vezes a gente deixa os materiais para eles construírem, entendeu?	25 Families, do they usually adhere to this strategy, or is it difficult for them to make themselves available to build together with you? GENILDA - Well, most of the time yes, many don't build for a reason like not having materials like scissors, because sometimes we leave the materials for them to build, you know?
ela não trazia brinquedo nenhum, ela trazia, às vezes quando tinha dificuldade, que o brinquedo era trabalhoso, ela já trazia pronta a atividade, na verdade, ou então a gente confeccionava junto, porque esse é um dos principais pontos. A gente não, na verdade as crianças confeccionavam, a gente só dava o auxílio. Eu falo pra Bruna, você estragou um monte de coisa, você estragou lençol, você estragou capa de sofá, meu chão era feio porque ele trazia tinta e o menino fazia a festa	26 she didn't bring any toys, she brought, sometimes when there was difficulty, when the toy was laborious, she already brought the activity ready, actually, or else we made it together, because that's one of the main points. We don't, actually the children make them, we just assist. I tell Bruna, you ruined a lot of things, you ruined sheets, you ruined sofa covers, my floor was ugly because he brought paint and the child had a party
Onde a gente tem mais dificuldade, como eu já retratei anteriormente, é quando a gente não leva material, sabe? Quando vai com proposta de brincadeiras, daquele guia mesmo que a gente recebe do Criança Feliz. Eu tenho até um... Ele aqui comigo. Ele é assim, não sei se você pode ver. Não sei se todos têm. Então, ele tem mais propostas de brincadeiras livres, que é interessante também. Mas eu acredito que, no momento, é mais difícil quando você vai sem material nenhum.	27 Where we have the most difficulty, as I mentioned earlier, is when we don't bring materials, you know? When we go with proposals for games, from that guide we receive from Happy Child. I even have one... It's here with me. It's like this, I don't know if you can see it. I don't know if everyone has it. So, it has more proposals for free play, which is also interesting. But I believe that at the moment, it is more difficult when you go without any material.
a gente não pode fornecer materiais que se compram na papelaria, e aí a gente também não pode exigir isso, porque as famílias nem sempre vão poder comprar, né, então, a ideia é que seja com o material que ela tem em casa, eu acho isso um limitador, eu acho que o próprio programa podia, poderia ter verba para isso.	28 we can't provide materials that are bought at the stationery store, and then we also can't demand that because families may not always be able to buy, right, so the idea is that it be with the material they have at home, I think this is a limiting factor, I think the program itself could, could have funds for this.
o que elas não aceitam muito bem é essa questão do método CDC ainda, porque foi sendo construído com elas, mas elas ainda sentem muita falta, porque elas construíam fantoches, construíam o jogo da memória, construíam várias coisas e levavam para as famílias. E agora, com esse com esse método CDC, que é o Cuidado para o Desenvolvimento da Criança, apesar do estado do Rio ainda não ter sido capacitado, o município de Petrópolis decidiu já implementar esse método para poder já tem um tempinho que esse método foi, está sendo implementado para ser uma forma gradativa, tanto para as visitadoras quanto para as famílias para não não serem capacitados e falar assim, ó hoje vocês faziam e amanhã não pode fazer mais. Ter uma coisa um pouco mais leve, uma mudança gradativa para as famílias e para as visitadoras, para que o impacto não fosse muito grande**. Mas elas sentem falta até hoje, nas reuniões elas comentam que gostavam de se sentiam útil, que era a palavra que ela falou, para construir esses brinquedos e ela em casa pegava vários materiais, nas casas de parentes, padari, então elas se sentiam mais ativas, elas se sentiam mais presentes na execução do programa. Então isso elas sentem muita falta, de precisar fazer com que a atividade sem material nenhum**	29 what they don't accept very well is this issue of the CDC method still, because it was being built with them, but they still miss it a lot, because they used to make puppets, they used to make memory games, they used to make several things and take them to the families. And now, with this CDC method, which is Child Development Care, although the state of Rio has not yet been trained, the municipality of Petrópolis decided to already implement this method in order to have been a while since this method was, is being implemented to be a gradual way, both for the visitors and for the families, so that they are not trained and say, oh today you did and tomorrow you can't do it anymore. Have something a little lighter, a gradual change for families and visitors, so that the impact is not too great. But they still miss it today, in meetings they comment that they liked to feel useful, which was the word she used, to build these toys and she would take various materials at home, in relatives' homes, bakery, so they felt more active, they felt more present in the execution of the program. So they miss a lot, to need to make the activity without any material
essa é uma dificuldade que a gente tem, porque às vezes a criança, ela espera, já espera da visitadora que ela leve algo diferente para aquela brincadeira, né, então a própria criança espera algo diferente, né, daí a visitadora às vezes chega lá sem nada, né, a criança acaba que fica frustrada e não se dedica à atividade. Então, por isso que nessas visitas a gente sempre tenta levar, né, um material, uma atividade, um livro, uma proposta de alguma coisa para que ela possa estar desenvolvendo ali	30 this is a difficulty that we have, because sometimes the child, they expect, already expect from the visitor that she brings something different for that game, you know, so the child itself expects something different, you know, then the visitor sometimes arrives there with nothing, you know, the child ends up getting frustrated and doesn't dedicate himself to the activity. So, that's why in these visits we always try to bring, you know, a material, an activity, a book, a proposal for something so that she can develop there
a gente sempre pede que a família guarde os potinhos, as embalagens, as caixinhas ali, para que as meninas possam estar levando algumas propostas para eles. Mas, assim, as próprias visitadoras guardam essas embalagens em casa. Eles adquirem esse hábito de estar reciclando esse material para poder elaborar propostas para levar para aquelas famílias que a gente sabe que têm aquela dificuldade, que, como diz, não dão tanta importância ao programa	31 we always ask the family to save the little pots, the packaging, the little boxes there, so that the girls can bring some proposals to them. But, like, the visitors themselves keep these packages at home. They acquire this habit of recycling this material to be able to elaborate proposals to take to those families that we know have that difficulty, which, as they say, don't give so much importance to the program
E vamos ser realistas, temos lá no método 45 minutos, mas muitas vezes não dá, a gente não tem condições de ficar 45 minutos naquela casa para se deslocar para outra mais próxima, que é bem distante, para fazer novamente, e aí leva um tempão. E assim, o método CDC, as capacitações, belíssimas, bem elaboradas, tudo, mas muitas vezes não condiz com a nossa realidade. Então, a gente tem que estar fazendo algumas adaptações, porque, por exemplo, como eu já tinha falado antes, no nosso primeiro contato, tem as atividades que são relacionadas, vamos supor, ao material que aquela família tem naquela casa. Mas a gente chega em famílias que não tem nem onde sentar, que a família não tem nenhum pote para fazer aquela atividade. Então, a gente tem que pensar em atividades diferenciadas para ajudar na explicação daquela família	32 And let's be realistic, we have there in the method 45 minutes, but often it doesn't, we don't have the conditions to spend 45 minutes in that house to move to another closer one, which is quite far, to do it again, and then it takes a long time. And so, the CDC method, the train
eu achava legal se eles tivessem uma parte visual das atividades que tem que fazer com o livro, porque às vezes não fica muito claro	33 I thought it would be nice if they had a visual part of the activities that they have to do with the book, because sometimes it is not very clear

Enabling Environment	English Translation
que o único recurso que está vindo certinho é o Crianças Felizes, os outros, infelizmente, o CRAS está falido, superfalido. Hoje quem sustenta um CRAS é o programa Crianças Felizes, porque é um recurso que vem todo mês e vem certinho, e vem um valor alto para nós, para nós é alto, é o que vem mais.	14:7 The only resource that is coming correctly is Happy Children, the others, unfortunately, the CRAS is bankrupt, very bankrupt. Today, what sustains a CRAS is the Happy Children program because it is a resource that comes every month and comes on time, and it comes at a high value for us, for us it is high, it is the most t comes
Então, infelizmente, ao longo desses anos, eu não senti ainda a esfera federal com a gente. A não ser esse compromisso de repassar os recursos, ainda bem que sempre foi muito correto, os meses que não deram certo, que teve a questão de cálculo de visita que eles fizeram errado, depois repassaram novamente, mas eles foram muito certinhos nesse sentido. Mas de acesso mesmo ao governo federal foi o mínimo.	14:12 So, unfortunately, over these years, I still haven't felt the federal sphere with us. Except for this commitm to pass on the resources, fortunately, it has always been very correct, the months that didn't work out, that had issue of miscalculating visits that they did wrong, they passed on again, but they were very correct in that sense But access to the federal government itself was minimal.
o município é pequeno, nós não temos vários funcionários numa mesma equipe. Por exemplo, a minha equipe tem quatro só assistentes sociais, só quatro, né? Então, para uma demanda de 18 mil hoje, muito pouco, né? Mas, é, nós não temos o censo. Olha outro absurdo, gente, como é que a política social, ela se reprograma sem um censo? Quer dizer, quando eu passo a informação para o Estado de que eu tenho, né, x necessidade por x famílias, o Estado me questiona, porque o censo que ele tem lá é de 2010.	11:5 The municipality is small, we don't have several employees in the same team. For example, my team has only four social workers, only four, right? So, for a demand of 18 thousand today, very little, right? But, yeah, w don't have the census. Look at another absurdity, folks, how is it that social policy, it reprograms itself without a census? I mean, when I pass on the information to the State that I have, right, x need for x families, the State questions me, because the census they have there is from 2010.
Então, acho que recurso devia melhorar, a principal, se a gente não tiver dinheiro, a gente não consegue executar. Ainda me intriga, na verdade, a criança ser hoje prioridade absoluta e o programa ainda depender da adesão do prefeito municipal. Sorte que o nosso aqui já havia colocado até no plano de trabalho dele, né.	8:2 So, I think the resource should improve, the main one, if we don't have money, we can't execute. It still intrigues me, in fact, that the child is now an absolute priority and the program still depends on the adherence o the municipal mayor. Luckily, ours here had already put it in his work plan, right.
É o retrato da má administração deste governo que só na política social, ele desmontou nossa política social, ele desmontou, tirou o ministério, reduziu em 40% todas as verbas e coibiu uma série de processos. Nós não temos, no governo federal, nenhum apoio, nenhum suporte e temos, ao contrário, além de uma verba reduzida, além do desmonte da política pública, o não reforço aos suas, ou seja, ele trincou os suas, deixou em pedaços, não integrou, não integrou, um ministério de cidadania que pouco entende, pouco importa, e tanto que os suas hoje está precisando de uma revisão, com urgência, porque ele conseguiu, em pouquíssimo tempo, dividir, diminuir o sistema único de assistência social, a começar diminuindo um recurso que já era pouco.	11:3 It is a reflection of the mismanagement of this government that only in social policy, it dismantled our socia policy, it dismantled, removed the ministry, reduced all budgets by 40%, and hindered a series of processes. W have, in the federal government, no support, no support, and we have, on the contrary, in addition to a reduced budget, in addition to the dismantling of public policy, the non-reinforcement of its, that is, it cracked its, left it in pieces, did not integrate, did not integrate, a Ministry of Citizenship that understands little, matters little, and so much so that the its today need a revision urgently, because he managed, in a very short time, to divide, dimini the single system of social assistance, starting with reducing a resource that was already small.
4 mil para você pagar servidor, custear o programa, manter o programa é muito complicado, então, aqui a gente ainda consegue, porque a prefeitura, o prefeito, ele, em relação à INSS, né, aquelas outras obrigações já são custeadas pela prefeitura, né, algumas despesas que a gente não consegue custear com recurso federal, a prefeitura acaba assumindo, aí a gente tem conseguido mais, se a gente tivesse mais recursos, seria muito melhor para a gente trabalhar o programa, né, porque o programa aqui no nosso município, ele ganhou uma proporção e que a gente precisa, é, a cada vez mais estruturar, né, tá estruturando, é, talvez, talvez não, é, com a procura que a gente já tem, né, que vai precisar de mais visitadores, né, e aí com o recurso mensal que a gente recebe é insuficiente ainda.	14:19 4 thousand to pay for staff, cover the program, maintain the program is very complicated, so, here we stil manage because the city hall, the mayor, in relation to INSS, right, those other obligations are already borne by the city hall, some expenses that we cannot cover with federal resources, the city hall ends up assuming, so we have achieved more, if we had more resources, it would be much better for us to work on the program, right, because the program here in our municipality, it has gained a proportion and that we need, it is, increasingly structuring, right, it is structuring, maybe, maybe not, it is, with the demand that we already have, right, that will need more visitors, right, and then with the monthly resource that we receive is still insufficient.
Mas acredito que essa foi a nossa nossa maior dificuldade a gente tinha vários e várias coisas planejadas para fazer, mas aí uma viagem passou 25 dias a outra vez passa 20 aí a gente não consegue se reunir nem pelo WhatsApp porque ainda tem um fator que nos municípios não pela internet. Aí você chega, você fica incomunicável	9:12 But I believe that this was our greatest difficulty we had several things planned to do, but then a trip passe 25 days the other time it passed 20 then we can't even meet by WhatsApp because there is still a factor that in municipalities not by the internet. So you arrive, you become incommunicado.
Então, a gente tem nossos nativos que moram aqui há muito tempo, famílias que a gente já acompanha há muitos anos, mas também a gente tem as famílias que estão vindo todo o tempo, estão saindo todo o tempo. Então, essa lista era muito volúvel. Então, vinha uma lista, aí, vamos atender essa, essa? Vamos lá? Quando a gente começava a atender, a família ia embora. Então, foi tudo num balaio só. Foi o desafio de você mostrar o programa no município, o desafio de você fazer a busca ativa dessas famílias do Bolsa Família e fazer também com que o programa funcionasse.	14:2 So, we have our natives who have been living here for a long time, families that we have been following fo many years, but we also have families that are coming all the time, they are leaving all the time. So, this list was very volatile. So, a list came, then, are we going to serve this, this? Let's go there? When we started serving, th family left. So, it was all in one basket. It was the challenge of showing the program in the municipality, the challenge of actively searching for these Bolsa Família families and also making the program work.
Na minha avaliação, a dificuldade do prefeito aderir, porque é opcional, né, não é uma coisa instituída, como foi pelo SUAS, CRAS e CREAS, né, é exatamente o pequeno recurso do programa e a contrapartida do município de arcar com a outra parte do programa, né. Então, eles, alguns, alguns entendem que, como eles têm creche, pré-escola, etc., Eles acreditam que possa estar, através desses meios, creches, pré-escola, estar inserindo pontos fundamentais do programa, sem a necessidade de implantá-lo, né.	11:1 In my assessment, the difficulty of the mayor's adherence, because it is optional, right, it is not an institute thing, as it was by SUAS, CRAS, and CREAS, right, it is exactly the small resource of the program and the counterpart of the municipality to bear the other part of the program, right. So, they, some, some understand th as they have daycare, preschool, etc., They believe that through these means, daycares, preschools, they may inserting fundamental points of the program, without the need to implement it, right.
A gente insere hoje uma criança no cadastro único, ela vai demorar de 3, de 30 a 90 dias para aparecer no meu sistema do IPCF. ENTREVISTADORA CAROL: Mas isso é um problema com todas as faixas etárias, porque isso já ouvi sendo relatado principalmente com recém-nascidos.GESTOR ANTONY: É, recém-nascido.	8:3 We enter a child into the Single Registry today, it takes from 30 to 90 days for it to appear in my IPCF syste INTERVIEWER CAROL: But is this a problem with all age groups because I've heard it being reported mainly w newborns. MANAGER ANTONY: Yes, newborns.
quando a gente insere uma família no CadeÚnico, a gente vai lá, o posto viu que tem uma criança na casa que tem a idade para o programa, manda para a gente, a gente vai inserir ela no Cadastro Único, demora 90 dias para o sistema fazer a leitura e considerar se a criança é parte do CadeÚnico. Então isso também já demora para nós, porque a gente já está com a família ali no gatilho, a gente está ali já pronto para atender, mas não dá para atender porque a gente até atende, tem umas que a gente atende, mas não contabiliza como visita, e acaba criando uma expectativa. Hoje eu vejo que o maior problema dentro do Criança Feliz é esse.	14:8 When we enter a family into the Single Registry, we go there, the office saw that there is a child in the hou who is eligible for the program, sends it to us, we will enter it into the Single Registry, it takes 90 days for the system to read and consider if the child is part of the Single Registry. So, this also takes time for us because we already have the family there on standby, we are already ready to assist, but we cannot assist because even though we do assist, some we do assist, but it does not count as a visit, and it ends up creating an expectation. Today I see that the biggest problem within Happy Children is this.
a gente teve a troca da equipe, né, que acabou seus contratos com as visitadoras, então foi necessário, entrou as seis novas visitadoras. E aí essa é a outra que não podia mais fazer parte da equipe como visitadora, a gente contratou ela para fazer essa parte de supervisão, orientação, que ali era uma das coisas que eu via que necessitava muito, né. Às vezes, ali no momento de criar uma atividade, de algum problema, alguma situação ali que as visitadoras tinham, né, sempre necessita de ter uma pessoa ali para dar uma orientação para elas.	18:95 we had the team change, right, which ended their contracts with the visitors, so it was necessary, six new visitors came in. And then this is the other one that could no longer be part of the team as a visitor, we hired he do this part of supervision, guidance, which was one of the things that I saw was very necessary, right. Sometimes, there at the moment of creating an activity, some problem, some situation there that the visitors ha right, always requires having someone there to give guidance to them.
quando chegou a questão das viagens, eh o setor do financiamento do conselho do fundo estadual ele diz o seguinte: Kelly voce tem X valor empenhado do teu recurso para essas viagens, tipo, 21 mil. Então, eu tenho 200 e 50 mil, mas para viagem eu só tinha 21 mil. Por isso que das 17 viagens que no início do ano eu planejei eu reduzi para nove, porque a gente ainda tem que calcular essa questão dos valores das viagens. Dentro desse valor de 21 mil já tinham empenho. Entendeu? Essa aí eu já passei, já passei para as secretárias para seguir o fluxo e fazer essas aquisições.	9:10 when it came to the issue of trips, the financing sector of the state fund council says the following: Kelly, y have X amount committed from your funding for these trips, like, 21 thousand. So, I have 250 thousand, but for the trip I only had 21 thousand. That's why of the 17 trips that I planned at the beginning of the year, I reduced to nine because we still have to calculate this issue of the trip costs. Within this 21 thousand already had a commitment. Understand? This I already passed on, already passed on to the secretaries to follow the flow and make these acquisitions.
Porém, eu acho que tem que ser um planejamento junto com o coordenador estadual para a gente ver a melhor maneira, porque as comunidades são bem distantes, são muitas comunidades, são mais de 20 aldeias. Há uma população indígena bem grande aqui do município de Jacareacanga e a maioria, vamos botar um 80% das crianças do cadastro único aqui do município fazem, são os indígenas. E aí seria importante, muito importante, acredito, mas dessa extensão do programa para as comunidades indígenas. Por enquanto ela está focada mais só aqui na cidade, né? A gente atende os indígenas aqui da cidade, não os da aldeia, por conta dessa logística de viagens, de que teria que ter uma equipe maior, entendeu? Para estar trabalhando e tudo mais.	15:6 However, I think there has to be planning together with the state coordinator to see the best way because communities are very distant, there are many communities, there are more than 20 villages. There is a very larg indigenous population here in the municipality of Jacareacanga and the majority, let's say 80% of the children in the single registry here in the municipality are indigenous. And so it would be important, very important, I believe but this extension of the program to indigenous communities. For now, it is focused more only here in the city, right? We serve the indigenous people here in the city, not those from the village, because of this travel logistic that there would have to be a larger team, understand? To be working and all.
eu penso que o recurso daqui não é que a gente queira ser melhor que os outros municípios, né, mas pensando no nosso território, eu penso que o nosso recurso deveria ser de acordo com o nosso território. Porque tem municípios que, às vezes, tem um público menor que o nosso, mas que não tem, aliás, o município às vezes tem a mesma quantidade de usuários que nós temos, e recebe o mesmo valor de recurso que nós recebemos. Só que se a gente for analisar onde essas famílias estão morando, né, é muito, assim, é desproporcional, porque eu tenho famílias aqui que moram a quase 80 quilômetros, é mais fácil eu chegar na capital do que eu chegar até essas famílias.	14:23 I think the resource here is not that we want to be better than other municipalities, right, but thinking abou our territory, I think our resource should be according to our territory. Because there are municipalities that sometimes have a smaller population than ours, but do not have, in fact, the municipality sometimes has the sa number of users as we have, and receives the same amount of resources as we receive. However, if we analyz where these families are living, right, it's very, like, it's disproportionate because I have families here that live almost 80 kilometers away, it's easier for me to get to the capital than to get to these families.
eu ainda acho que a comunicação, a divulgação é o que, entre os profissionais, a conscientização e divulgação para os profissionais de todas as áreas, né? Para que eles possam, quando identificar um caso possível, direcionar seja para saúde, para educação, mas de certa forma que a gente consiga fazer chegar no programa, né? No PCF.	7:5 I still think that communication, dissemination is what, among professionals, awareness and dissemination t professionals from all areas, right? So that they can, when identifying a possible case, direct it either to health, education, but in a way that we can manage to get to the program, right? In PCF.
e principalmente os desafios que a gente tem aqui nos nossos municípios, que são municípios pequenos, que eles têm uma população muito mais carente, né, que a gente enfrenta bastante desafios aí.	18:93 and especially the challenges that we have here in our municipalities, which are small municipalities, whic have a much more needy population, right, that we face quite a few challenges there.

Monitoring

Perguntei se podia manter o registro só no IPCF, que é o sistema que registra as visitas. Mas eu também fui negado. Por enquanto, tem que preencher os planos, as folhas e tudo. Eu falei, é muito papel. Muito papel. A gente também não tem mais arquivo para guardar tanto papel aqui no CRAS.

Aqueles materiais a gente, agora, por que eu falo agora? Porque foi difícil adaptarmos a ele, porque parece simples, mas não é. Para preencher todas aquelas fichas, são várias perguntas, são muitas perguntas, acho que o mais fácil é do território, da criança são perguntas, até às vezes que eu acho uma das perguntas desnecessárias, se a criança tem certidão de nascimento, como é que ela vai estar dentro de um programa se ela não tiver certidão de nascimento, né? Então, assim, são várias perguntas. E no começo, para responder, fazer aquilo tudo ali, demorou um pouco, **e o mais demorado foi, acho que é o de verificação, o desenvolvimento, aquela ficha que pergunta, faz sozinho, faz com ajuda, ou não consegue fazer? De zero a três meses, de novo, a criança consegue subir de grau, faz sozinho, faz com ajuda, ou não consegue fazer? Essa ficha em si foi a mais demorada para aprender, foi mais de ano para a nossa equipe entender que aquilo era importante. Para eu passar essa informação, para que eles entendessem que aquilo realmente** de fato era importante, foi complicado. Por quê? Porque achavam desnecessário

Agora a coleta de dados também é sempre um desafio, né? Porque a gente tem esse... Nós somos esse município com uma característica... Existe um nome próprio para isso? Deixa eu ver se eu me lembro... Não. Mas enfim, muitas pessoas que mudam para cá ficam dois, três, quatro meses, depois vão embora. Sabe, é muita migração aqui dentro.

antes dessa portaria de 2019, que fala que os estados têm que fazer monitoramento do programa, o estado de São Paulo já se antecipou fazendo contratação da FESP, para que eles nos apoiassem a fazer esse monitoramento. Então, a gente contratou uma universidade que rodou alguns municípios, enfim, criaram alguns instrumentos para a gente de monitoramento, e aí, isso em 2018, e aí em 2019, e aí em 2019, vem o Ministério com dois instrumentos prontos para a gente aplicar no território, sendo que a gente já tinha o nosso, então a gente já se antecipou nesse sentido. Então, a gente tem uma plataforma de monitoramento do programa, a gente fez isso em conjunto com o Ministério

para elas nos demandarem situações específicas, às vezes do próprio colega do que está trabalhando junto e na reunião em dupla não poderia falar, situações que ela encontra na casa da pessoa que está trabalhando. Situações que ela encontra na casa da pessoa e ela não pode comentar em grupo, ela só pode só pode comentar conosco, que nós somos supervisores, situações de agressão, situações de violência, situações de negligência

e aí a gente acaba deixando alguma coisa de lado para fazer essas reuniões e a gente entende que as reuniões são importantes, mas todo o restante também é, então a gente... Eu acho que isso é um dificultador, porque quando você perde um pouquinho esse vínculo, essa confiança do visitador com o supervisor, a gente passa a não ter tanta ligação com a família, porque a gente só tem a ligação com a família através dos visitadores.

Voltando lá para questão dos novos servidores, quando você fala sobre as demandas, né, eles eles precisam, . A gente percebe que eles precisam dessa capacitação, porque conversando com outros visitadores antigos, eles sempre falam que os novatos estão perdendo a essência do programa, e essa questão do... eles têm um grupo, . Um grupo de supervisores e eles passam muitas fotos, muitos vídeos, mas a gente percebe que ainda falta aquela aquela temática principal, cuidador, atividade com cuidador, criança, entendeu?

agora com o sistema EPCF, eu acho que vai ter que adequar essa capacitação, porque vai ter que ter um dia só de sistema, porque o sistema, a Amanda pode falar melhor que eu, o sistema reflete totalmente a qualidade do programa no município, seja olhando para as visitas, seja olhando para as equipes, e principalmente porque reflete no financiamento,

Mas acho que apresentar dados mais estatísticos mesmo, né? Trabalhar com isso seria mais... A gente vê mais resultado, né? Quando acaba mexendo com números, né? E nós sabemos, a gente sempre fala que o nosso resultado são essas vidas que a gente toca e você vê diferença, né?

English Translation

15:15 I asked if it was possible to keep the record only in IPCF, which is the system that records the visits. But I was also denied. For now, we have to fill out the plans, the sheets, and everything. I said, i too much paperwork. A lot of paperwork. We also don't have space anymore to store so much paper h at the CRAS.

7:01 Those materials, now, why do I say now? Because it was difficult for us to adapt to it, because it seems simple, but it's not. To fill out all those forms, there are several questions, many questions, I thi the easiest ones are about the territory, about the child, there are questions, sometimes I find some of the questions unnecessary, like if the child has a birth certificate, how will they be in a program if they don't have a birth certificate, right? So, there are several questions. And in the beginning, to answer, t do all that there, it took a while, and the longest was, I think it's the verification, the development, that form that asks, does it do it alone, does it do it with help, or can't do it? From zero to three months, ag can the child move up a grade, does it do it alone, does it do it with help, or can't do it? This form itself was the slowest to learn, it took our team more than a year to understand that it was important. For me pass on this information, for them to understand that it really was important, it was complicated. Why? Because they thought it was unnecessary.

7:2 Now data collection is always a challenge, right? Because we have this... We are this municipality with a characteristic... Is there a proper name for it? Let me see if I remember... No. But anyway, many people who move here stay for two, three, four months, then leave. You know, there's a lot of migratio here.

7:03 Before this 2019 ordinance, which states that states have to monitor the program, the state of Sã Paulo anticipated this by hiring FESP to support us in monitoring. So, we hired a university that went through some municipalities, created some instruments for us for monitoring, and so, this was in 2018, and then in 2019, and then in 2019, the Ministry comes with two ready instruments for us to apply in th territory, whereas we already had ours, so we already anticipated in this sense. So, we have a progra monitoring platform, we did this together with the Ministry.

16:5 We also had individual meetings because individual meetings are extremely important for them to demand specific situations, sometimes about the colleague they are working with and couldn't speak i the pair meeting, situations they encounter at the person's house they are working with. Situations they encounter at the person's house and they can't comment in the group, they can only comment with us, we are supervisors, situations of aggression, situations of violence, situations of neglect.

16:6 We have a lot of things to do, reports to read, visit plans to follow up on, and then we end up leav something aside to have these meetings, and we understand that the meetings are important, but everything else is also, so we... I think this is a hindrance because when you lose a little bit of that connection, that trust of the visitor with the supervisor, we no longer have so much connection with the family because we only have the connection with the family through the visitors.

9:4 Going back to the issue of new employees, when you talk about the demands, right, they need it. W realize that they need this training because talking to other experienced visitors, they always say that t newcomers are losing the essence of the program, and this issue of... they have a group, a group of supervisors and they send many photos, many videos, but we realize that there is still a lack of that ma theme, caregiver, activity with caregiver, child, you know?

9.5 Now with the EPCF system, I think we will have to adjust this training because there will have to be day just for the system because the system, Amanda can speak better than me, the system totally refle the quality of the program in the municipality, whether looking at the visits, looking at the teams, and mainly because it reflects in the funding.

7:4 But I think presenting more statistical data, right? Working with it would be more... We see more results, right? When it ends up involving numbers, right? And we know, we always say that our result i these lives that we touch and you see a difference, right?

Program Design	English Translation
mas uma pessoa auxiliando é mil vezes melhor. Então eu mesmo, pra mim, esse programa, se eu pudesse, todo ano eu tenho um filho, nunca saio desse programa.	3:3 but having someone assisting is a thousand times better. So, for me, this program, if I could, every year I would have a child, I never leave this program.
E a questão do zero a três anos, que eu acho que já passou da hora da gente ter um outro olhar, de estender mais para zero a seis, até porque vai falar de serviço de convivência de zero a seis anos, o que nós vamos fazer de serviço de convivência com zero a seis anos dentro de um caso? Hoje não tem mais, então o Criança Feliz ia conseguir acolher essa demanda para nós, que é uma demanda emergente	14:10 And the issue of zero to three years old, which I think it's time for us to have a different look, to extend it more to zero to six, also because it will talk about coexistence services from zero to six years old, what are we going to do with coexistence services for zero to six years old within a case? Nowadays, it doesn't exist anymor so Criança Feliz would be able to meet this demand for us, which is an emerging demand
O desafio também, a gente pode ter o desafio também de quando a criança vai se desligar também no programa, aos três anos que a gente tanto busca para, entendendo que a primeira infância é até os seis anos, por que não estender mais, por que até os três anos?Então, também é um desafio para a gente parar aí, praticamente no meio do caminho	19:8 The challenge also, we might have the challenge of when the child is going to be disconnected from the program, at three years old that we are so eager for, understanding that early childhood is up to six years old, w not extend it more, why only up to three years? So, it's also a challenge for us to stop there, practically in the middle of the way
A compra de bicicleta, não entendo por que não pode, não entendo mesmo, motocicleta, o carro nosso, eu tive que juntar dois anos de recurso. Então, fiz uma expectativa de juntar um dinheiro todo mês e, no final desses dois anos, a gente conseguiu comprar um carro para nós. É um carro que é do Programa Internos Felizes, que, infelizmente, a gente não tem apoio da gestão. Ah, vou comprar um carro para o Social. O Social vai ficar com o carro que não está servindo mais para ninguém. Mas é o que todo mundo tem que atender. A gente tem que atender todo mundo, mas o nosso carro sempre é o pior. Então, eu falei, não, vamos comprar um carrinho bonitinho, nós demoramos dois anos para comprar, compramos um carro bom e hoje a gente tem o apoio do carro. Mas essa questão da bicicleta também não consigo entender por que é proibido, da motocicleta também não consigo entender	14:14 The purchase of a bicycle, I don't understand why it's not allowed, I really don't understand, motorcycle, o car, I had to save for two years. So, I made an expectation to save money every month and, at the end of these two years, we managed to buy a car for us. It's a car that belongs to the Happy Interns Program, which unfortunately, we don't have support from management. "Oh, I'll buy a car for Social." Social will end up with the car that's no longer serving anyone. But that's what everyone has to comply with. We have to comply with everyone, but our car is always the worst. So, I said, no, let's buy a cute little car, it took us two years to buy it, w bought a good car and today we have the support of the car. But this issue of the bicycle I also can't understan why it's prohibited, neither the motorcycle.
trabalhou essa criança, cadastrou, perto de chegada do período para entrar no meu IPCF, o que aconteceu com essa família?ENTREVISTADORA CAROL: Foi embora.GESTOR ANTONY: Já foi embora. Então a gente perdeu o tempo.ENTREVISTADORA CAROL: É um desafio, Antony.GESTOR ANTONY: Perder o tempo não, né?ENTREVISTADORA CAROL: É um desafio porque você pensando na quantidade de famílias que é perfil, que também podem ser atendidas e...GESTOR ANTONY: E a gente quer atender essa criança, quer fazer com que o trabalho funcione, e ao mesmo tempo a gente não tem essa agilidade nos nossos sistemas, por exemplo. Não temos essa agilidade. Poxa, a família bateu alguém na porta do CRAS, a gente consegue ir praticamente na mesma semana e atender ela. O problema é que eu não vou conseguir nem receber o recurso porque não vai estar no período. Então, é uma dificuldade nossa, é um desafio	8:5 this child worked, registered, close to the time to enter my IPCF, what happened to this family? INTERVIEWER CAROL: They left. MANAGER ANTONY: They already left. So we lost time. INTERVIEWER CAROL: It's a challenge, Antony. MANAGER ANTONY: Not losing time, right? INTERVIEWER CAROL: It's a challenge because when you think about the number of families that fit the profile, that could also be served an MANAGER ANTONY: And we want to assist this child, we want to make the program work, and at the same tim we don't have that agility in our systems, for example. We don't have that agility. Gosh, a family knocks on the CRAS door, we can practically go there the same week and assist them. The problem is that I won't even be ab to receive the funding because it won't be in the period. So, it's our difficulty, it's a challenge.
E pro Oliver, eu achei legal semanal, porque eu percebi que o desenvolvimento dele agora é semanal,	5:49 And for Oliver, I found it nice weekly, because I noticed that his development is now weekly.
eu achei legal a proposta de a visita não ser toda semana presencial. Eu achei bom essa proposta, por quê? Porque, sabe aquelas diaristas que fazem diária de 15 em 15 dias? Elas agora podem participar, que elas não podiam participar, elas agora podem	15:14 I found the proposal of the visit not being every week in person good. I found this proposal good, why? Because, you know those daily maids who work every two weeks? Now they can participate, they couldn't befo they can now.
eu achava legal se eles tivessem uma parte visual das atividades que tem que fazer com o Oliver, porque às vezes não fica muito claro	5:50 I thought it would be nice if they had a visual part of the activities they have to do with Oliver because sometimes it's not very clear.
eu acho que é pouco, porque e acho que em 15 minutos uma criança não mostra a dificuldade dela, tá certo que quem tem que acompanhar é os pais, só que é como eu falei desde o começo, um olhar de outra pessoa ajuda mais, então assim, não também pegar o dia todo de uma visitadora que também cada um tem suas coisas pra fazer, mas estabelecer um tempo que dê pra todo mundo, sim, tá bom, estabelecer um horário que dê assim pra ajudar tanto a mãe quanto a visitadora seria melhor, claro, pra ajudar um pouco mais	3:1 I think it's too little because I think in 15 minutes a child doesn't show their difficulty, it's true that parents are the ones who have to monitor, but as I said from the beginning, another person's perspective helps more, so, n taking the whole day from a visitor who also has their own things to do, but establishing a time that works for everyone, yes, that's good, establishing a time that works for both the mother and the visitor would be better, of course, to help a little more
É muito rápido,né? Porque ela não faz atividade com a gente, ela só entrega o que se tem que fazer e vai embora. Aí a gente conversa um pouco, ela pergunta como é que tá, tira foto com ele, e ela vai embora. É rapidinho. Eu acho rapidinho	5:46 It's very quick, right? Because she doesn't do activities with us, she just hands over what needs to be done and leaves. Then we talk a bit, she asks how he's doing, takes a photo with him, and she leaves. It's quick. I fin quick.
Era um tempo ideal, elas ficavam aqui entre 20 e meia hora, né? Ficando aqui conversando, mexendo com ela para ela poder despertar um pouquinho mais de interesse	6:1 It was an ideal time, they stayed here for about 20 to 30 minutes, right? Staying here talking, interacting wit her so she could become a bit more interested.
mas eu gostaria que fosse mais tempo delas passando com as crianças, porque elas realmente têm muito o que ensinar para as crianças, só que o tempo é muito curto, porque às vezes o tempo é pouco para ter que atender todas as crianças, né?	6:3 but I would like it to be more time spent with the children because they really have a lot to teach the childre it's just that the time is too short because sometimes the time is not enough to attend to all the children, right?
ele não foi pensado na característica do município, né, igual aquele assunto das 45 minutos da visita. O que esperava-se, né, acho que quando criou-se o programa, que uma casa fosse do lado da outra, que o visitador conseguisse atender, hoje cada visitador nosso atende 25 famílias, que é a meta deles, cinco famílias no dia seria. Esperava-se que essas famílias morassem todas pertas, imagino, né, para dar o tempo necessário para as visitas, mas não funciona desse jeito	8:1 it wasn't designed for the municipality's characteristic, right, like that issue of the 45-minute visit. What was expected, right, I think when the program was created, was that one house would be next to another, that the visitor could assist, today each of our visitors assists 25 families, which is their goal, would be five families per day. It was expected that these families all lived close, I imagine, to give the necessary time for the visits, but it doesn't work that way
Uma visita domiciliar de 45 minutos para todas as famílias aqui para a gente, se fossem de 40 horas os trabalhadores, daria em média até 37 horas semanais. E já ia comprometer pelo deslocamento, tornando-se até inviável cumprir esse tempo na visita.	15:8 A home visit of 45 minutes for all families here for us, if the workers had 40 hours, would average up to 37 hours per week. And it would already compromise due to the commute, becoming even unfeasible to meet this time in the visit.
Porque não é todo mundo que aceita pessoas dentro da sua casa, né? Então o entrar em casa, a gente tem que conscientizar muito essa mãe do quanto é importante. Tentar realmente criar esse vínculo é um grande desafio. Eu acho que talvez seja o maior desafio para a entrada na casa, né	7:1 Because not everyone accepts people into their homes, right? So entering the house, we have to really rais awareness of this mother about how important it is. Trying to really create that bond is a big challenge. I think maybe it's the biggest challenge for entering the house, right?
Então, a gente, depois a gente começou a usar de forma híbrida também, mas eu vou falar para vocês, o modo remoto, quando a gente recebe um retorno, a gente fica muito feliz, a gente chega até a comemorar, porque é muito complicado. Para a gente não funciona esse remotamente, gente, me desculpem.	15:12 So, we, later on, we started using it in a hybrid way too, but I'll tell you, the remote mode, when we receiv feedback, we are very happy, we even celebrate, because it's very complicated. For us, it doesn't work remotel folks, I'm sorry.

Training	English Translation
e seria bom assim para a gente, nesse momento se pudesse ofertar como lidar com o público, nós somos tímidos. Então, essa parte para tá a frente, para perder o medo, para falar seria bastante interessante. Eu não sei se vocês trabalham nesse segmento, mas aí seria até uma sugestão também, que eu acho que iria enriquecer também nesse momento	9:3 and it would be good for us, at this moment, if we could offer how to deal with the public, we are shy. So, th part to be ahead, to lose the fear, to speak would be quite interesting. I don't know if you work in this segment, it would also be a suggestion, which I think would enrich at this moment.
Voltando lá para questão dos novos servidores, quando você fala sobre as demandas, né, eles eles precisam, . A gente percebe que eles precisam dessa capacitação, porque conversando com outros perdedores antigos, eles sempre falam que os novatos estão perdendo a essência do programa, e essa questão do... eles têm um grupo, . Um grupo de supervisores e eles passam muitas fotos, muitos vídeos, mas a gente percebe que ainda falta aquela aquela temática principal, cuidador, atividade com cuidador, criança, entendeu?	9:4 Going back to the issue of new servers, when you talk about the demands, right, they need it. We realize th they need this training because talking to other old supervisors, they always say that the newcomers are losing the essence of the program, and this issue of... they have a group. A group of supervisors and they share man photos, many videos, but we notice that the main theme is still missing, caregiver, caregiver activity, child, you know?
ENTREVISTADORA CAROL: Você teve alguma capacitação em relação às metodologias do programa?GESTORA AMANDA: Não.	12:67 INTERVIEWER CAROL: Did you have any training regarding the program methodologies? MANAGER AMANDA: No.
Acho que tinha uma cartilha, se não me engano, na época que só trazia um esboço do que a gente tinha que fazer. Nós nos reunimos com elas. Infelizmente, essa questão dos visitadores sempre foi um grande problema dentro do programa, porque a capacitação para eles... Nunca houve uma capacitação tête-à-tête, presencial, era sempre o supervisor, ou o gestor, ou o coordenador, que tinha capacitação e trazia para o município. E aí, nós iniciamos a etapa de elaboração do que nós íamos faze	14:1 I think there was a booklet, if I'm not mistaken, at the time that only brought a sketch of what we had to do We met with them. Unfortunately, this issue of visitors has always been a big problem within the program becau the training for them... There was never a face-to-face training, it was always the supervisor, or the manager, o the coordinator, who had training and brought it to the municipality. And then, we started the stage of elaborati what we were going to do
a gente convidar um especialista, um pedagogo mesmo, né, para a gente fazer um, assim, uma programação de metade de um dia ou de um dia inteiro, porque quando a gente vai buscar coisas, né, o conhecimento, e a formação base, por exemplo, né, social interativismo, cognição, a coisa motora, visual, motora fina, imagina alguém que não é da área tentando entender esses termos, né, ou você faz pergunta, ou vai para o dicionário, ou vai pesquisando outros artigos	16:8 we invite a specialist, a pedagogue, right, for us to do, like, a half-day or a full-day program, because whe we go searching for things, right, knowledge, and basic training, for example, right, social interaction, cognition, motor thing, visual, fine motor, imagine someone who is not from the field trying to understand these terms, righ either you ask a question, or you go to the dictionary, or you search for other articles.
Eu gostaria de uma capacitação não sei todos os meninos que só tem 6 meses de questão de violência contra as crianças, porque aqui em petrópolis aconteceu de uma menina de 4 anos ser espancada e morreu então eu gostaria de ter uma capacitação que fosse específica	19:3 I would like a training, I don't know if all the guys who only have 6 months of violence against children, because here in Petrópolis it happened that a 4-year-old girl was beaten and died, so I would like to have a specific training for this.
a capacitação, eu acho que só teria que ter mais uma capacitação em questão desse BPC. As crianças mas esse olhar pra gente conseguir entender mais essa capacitação das crianças que são ou deficientes ou que tem alguma dificuldade para fazer essa atividade.	19:1 the training, I think we just need to have one more training regarding this BPC. The children, but this look f us to understand more this training of children who are either disabled or who have some difficulty to do this activity.
que aí gera uma constante rotatividade dos profissionais...- Os municípios que aconteceu com as equipes como teve a troca de gestão, não trocou só um supervisor e um visitador. A maioria dos municípios dos nossos 24 foi a equipe toda. E assim ele sai, não ficavam para passar para o outro, entendeu? Acabou, acabou, então aí por isso que a gente ficou meio perdidos. CAROLINA - É como se houvesse uma descontinuidade do serviço, não é isso? - Isso	9:6 which then generates a constant turnover of professionals... - The municipalities that happened with the tea as there was a change of management, it wasn't just one supervisor and one visitor who changed. In most of o 24 municipalities, it was the whole team. And so, they leave, they didn't stay to pass it on to the other, you kno It's over, it's over, so that's why we were kind of lost. CAROLINA - It's as if there was a discontinuity of service, isn't it? - That's it
a gente teve a troca da equipe, né, que acabou seus contratos com as visitadoras, então foi necessário, entrou as seis novas visitadoras. E aí essa é a outra que não podia mais fazer parte da equipe como visitadora, a gente contratou ela para fazer essa parte de supervisão, orientação, que ali era uma das coisas que eu via que necessitava muito, né. Às vezes, ali no momento de criar uma atividade, de algum problema, alguma situação ali que as visitadoras tinham, né, sempre necessita de ter uma pessoa ali para dar uma orientação para elas	18:95 we had the change of the team, right, which ended their contracts with the visitors, so it was necessary, s new visitors came in. And then this is the other one who could no longer be part of the team as a visitor, we hir her to do this part of supervision, guidance, which was one of the things that I saw that was very necessary, rig Sometimes, there in the moment of creating an activity, some problem, some situation there that the visitors ha right, it always needs to have someone there to give guidance to them
Primeiro que, se a gente está falando realmente da questão das estagiárias, a gente vai ter uma renovação de quadro, ou anual, ou a cada dois anos obrigatória. Então, vai ter que capacitar todo mundo novamente. Eu acho que uma capacitação continuada, ela é muito importante.	7:7 Firstly, if we are really talking about the issue of interns, we will have a renewal of the team, either annually every two years mandatory. So, we will have to train everyone again. I think ongoing training is very important.
vocês avaliam esse formato virtual, assim, que é que vocês acham? Você já trouxe essa questão de que na sua concepção ele é bem resumido mas como que você avalia assim? KELLY - ele é bem mais amplo mas assim como eu disse como a gente sempre conversa o online tem muita teoria dá para você ler, mas fazer uma leitura e ouvir de alguém no formato presencial é mais que enriquecedor, entendeu?	9:1 do you evaluate this virtual format, like, what do you think? You already brought up the issue that in your vi it's quite summarized but how do you evaluate it? KELLY - it's much broader but as I said as we always talk, online has a lot of theory you can read, but making a reading and hearing from someone in a face-to-face form is more enriching, you know?
mas eles queriam ela presencialmente. Então, para fazer um planejamento, para trazer pessoas de fora, que realmente trazem uma bagagem meio bacana para participar de uma capacitação presencial, tem que ter um recurso maior.	9:8 but they wanted it in person. So, to plan, to bring people from outside, who really bring a pretty good bagga to participate in face-to-face training, you need more resources,
a gente faz as capacitações necessárias, mas está tudo muito virtual. Então eu senti um pouco de diferença e dificuldade nisso, a gente tem que se esforçar muito mais para conseguir um aprendizado de fato.	16:1 we do the necessary training, but it's all very virtual. So I felt a bit of a difference and difficulty in this, we have to make much more effort to achieve actual learning.
Acredito que a capacitação presencial aí abrangeria muito mais detalhes, como eu trabalhava aqui na gestão e a antiga coordenadora, ela vinha, ela trazia n-experiências, n-relatos do que ela vivenciou nessas capacitações. Então, acredito que seja mais um pouco disso, né, dessas dificuldades.ENTREVISTADORA CAROL: Que esse formato virtual, ele, na sua opinião, ele compromete a qualidade do que é ofertado nas capacitações.SUPERV. LUCAS: Isso, né	18:102 I believe that face-to-face training would cover much more details, as I worked here in management and the former coordinator, she came, she brought n-experiences, n-reports of what she experienced in these trainings. So, I believe it's a bit more of that, right, of these difficulties. INTERVIEWER CAROL: That this virtual format, it, in your opinion, compromises the quality of what is offered in the trainings. SUPERV. LUCAS: That's right
até porque online o Ministério nem capacitou ninguém, eles disponibilizam esses cursos, EAD, mas nem tem capacitação online, assim, eles fizeram a do GVD, né, a do GVD online, mas não... A gente participou só da linha mesmo. É, a gente participou daquela... Eles fizeram o módulo do Guia da Visita numa plataforma separada, que é o AVA, pela Aliança Brasileira de Educação, mas não pegou muito, não, assim, eles reuniram as coordenações, a gente fez um alinhamento, deu uma olhada no conteúdo, mas os municípios ainda preferem isso daqui, a gente se reunindo e discutindo com eles, porque é diferente deles pegarem e fazerem um curso sozinhos, né	18:110 because even online the Ministry didn't train anyone, they provide these courses, EAD, but there's no online training, so, they did the GVD, right, the GVD online, but not... We only participated in the line itself. Yes we participated in that... They did the Visitor Guide module on a separate platform, which is the AVA, by the Brazilian Alliance of Education, but it didn't catch on much, no, they brought together the coordinations, we aligned, took a look at the content, but the municipalities still prefer this, us meeting and discussing with them, because it's different from them taking and doing a course alone, right.
é claro que a gente passa por todos os conteúdos, principalmente os formulários, a gente tem muita preocupação com os formulários, a gente percebe que os municípios têm muita dificuldade no plano de visita, por exemplo, em fazer o plano de visita, isso também vai repetir muito na qualidade, porque se você não planeja a visita, não é visita do criança feliz, eu brinco e falo que é visita de comadre, porque visita que não é planejada é visita de comadre, aquela que você chega de repente na casa da pessoa, sem avisar, sem saber nem o que você vai falar, então o criança feliz tem que ser planejado, a gente tem essa preocupação muito forte aqui no planejamento	Of course, we go through all the contents, especially the forms, we are very concerned about the forms, we no that municipalities have a lot of difficulty with the visit plan, for example, in making the visit plan, this will also repeat a lot in the quality, because if you don't plan the visit, it's not a Criança Feliz visit, I joke and say it's a vis like old friends dropping by, because a visit that is not planned is like an old friend's visit, one where you sudde arrive at someone's house, without warning, without even knowing what you're going to say, so Criança Feliz vi have to be planned, we have a very strong concern about this in our planning.

Workforce Expectations	English Translation
a gente teve a troca da equipe, né, que acabou seus contratos com as visitadoras, então foi necessário, entrou as seis novas visitadoras. E aí essa é a outra que não podia mais fazer parte da equipe como visitadora, a gente contratou ela para fazer essa parte de supervisão, orientação, que ali era uma das coisas que eu via que necessitava muito, né. Às vezes, ali no momento de criar uma atividade, de algum problema, alguma situação ali que as visitadoras tinham, né, sempre necessita de ter uma pessoa ali para dar uma orientação para elas	18:95 We had a team turnover, right, their contracts with the visitors ended, so it was necessary, six new visitor came in. And then this is the other one who couldn't be part of the team as a visitor anymore, we hired her to do this part of supervision, guidance, which was one of the things that I saw was much needed, you know. Sometimes, in the moment of creating an activity, of some problem, some situation that the visitors had, right, it always needs to have someone there to give them guidance
Eu acho que sim. Até porque assim, aqui na cidade existe muitos indígenas e, eu sou mestiça, sabe? A minha mãe é indígena, então eu não tenho tanta dificuldade em compreender, né? A língua materna. Mas conheço vários indígenas que reside aqui na cidade também, inclusive alguns que participa também desse programa, o programa criança feliz, que muitas vezes fala: “Ah eu não entendo, não sei como é que é, eu não sei o que significa”. Então eu acho que botasse um pouco mais, ajudar até mais pra língua portuguesa que é o que as crianças tem muita dificuldade por falar somente a língua … , que é a língua indígena diferente daqui, eu acho que seria interessante	4:50 I think so. Because here in the city, there are many indigenous people and, I'm mixed-race, you know? My mother is indigenous, so I don't have much difficulty in understanding, right? The native language. But I know several indigenous people who also live here in the city, including some who also participate in this program, th Criança Feliz program, who often say: "Oh, I don't understand, I don't know what it means". So I think putting a little more, helping even more with the Portuguese language, which is what the children have a lot of difficulty w speaking only the ... language, which is the indigenous language different from here, I think it would be interesting
Enfrentamos muitas dificuldades, porque nós temos aqui assentamento, temos quilombola, temos sem-terra, é muita gente para pouco recurso.	13:65 We face many difficulties because we have settlements here, we have quilombola communities, we have landless people, there are many people for few resources.
No entanto a gente tem até uma dificuldade na questão dos indígenas também que moram aqui na cidade do que a gente só atende os que moram aqui, a gente não tem famílias na aldeia pela conta da distância muito grande, e aí é uma dificuldade que a gente tinha com os indígenas que moram aqui na cidade que são muitos era na língua né, não falam muito bem português, e aí agora a gente tem uma visitadora que ela é bilíngue	15:1 However, we even have difficulty with the issue of indigenous people who live here in the city than we only assist those who live here, we don't have families in the village because of the great distance, and then it's a difficulty we had with the indigenous people who live here in the city, which are many, it was in the language, th don't speak Portuguese very well, and now we have a visitor who is bilingual
Aí foi quando o Antony, o Antony já nessa época, ele começou a assumir mais, porque veio uma portaria do Ministério que a gente tinha que ter um supervisor 40 horas ou dois de 20. Aí pegou para nós, né, veio a exigência, mas não veio recurso para pagar. Então, eu falei, puxa vida, e agora, né? A gente, vou tirar um assistente social para fazer o papel de supervisor, vou ter que contratar outro, e aí começou a pegar isso, não podia fazer contratação, veio uma especificação de que não podia ser terceirizado, tinha que ser ou da folha já, ou nomeação, foi daí que eu conversei com o prefeito, falei, prefeito, o senhor sabe já da importância que o programa está no município, né, e agora a gente já está se estendendo ao vale, ao estado, nós precisamos nomear um supervisor. Eu já acredito que o Antony está mais preparado. Aí foi quando ele recebeu uma nomeação, nós criamos um cargo para ele, existe um cargo de supervisor do programa Criança Feliz dentro dos cargos de comissão no município	14:4 Then it was when Antony, Antony already at that time, he started to take over more, because a decree ca from the Ministry that we had to have a supervisor for 40 hours or two for 20. Then it fell on us, right, the requirement came, but no funding came to pay. So, I said, wow, and now, right? We, I'll take a social worker to play the role of supervisor, I'll have to hire another, and then it started to catch on, I couldn't hire, there was a specification that it couldn't be outsourced, it had to be either already on the payroll or appointment, that's whe talked to the mayor, I said, mayor, you already know the importance of the program in the municipality, right, an now we are already extending to the valley, to the state, we need to appoint a supervisor. I already believe that Antony is more prepared. That's when he received an appointment, we created a position for him, there is a position of supervisor of the Criança Feliz program within the commission positions in the municipality.
Olha, a questão da contratação, porque isso dificulta muito os municípios, muito, muito, muito. Eu acho que deveria vir mais simplificado, porque eu sou uma gestora que eu fui atrás, mas a maioria dos cargos de gestão não são cargos políticos, que infelizmente, às vezes, caem na mão de pessoas que não têm preparação nenhuma. E o gestor faz a diferença, porque é o gestor que vai brigar, que vai pedir para o prefeito, que vai atrás de tudo dentro de um órgão. Então, acho que a questão de contratação tinha que ficar muito mais simplificado. Eles pioraram agora, porque eles estão colocando que a gente não pode mais contratar terceirizadas	14:13 Look, the issue of hiring, because this greatly complicates municipalities, a lot, a lot, a lot. I think it should be more simplified, because I am a manager who went after it, but most management positions are not political positions, which unfortunately sometimes fall into the hands of people who have no preparation at all. And the manager makes a difference because it's the manager who's going to fight, who's going to ask the mayor, who' going to go after everything within an agency. So, I think the hiring issue should be much simpler. They made it worse now because they are saying that we can no longer hire outsourced workers.
e às vezes a gente tem dificuldade, por exemplo, para, por exemplo, fazer um processo seletivo, eu preciso de um edital, as pessoas aqui do nosso município precisam entender também o que é um edital, o que é que nesse edital está solicitando, e assim, a gente tem, a grande maioria das, da nossa população aqui, é a formação em nível médio, né, então às vezes essa burocratização acaba complicando, para que a gente tenha, acaba contribuindo para que a gente tenha essas dificuldades	14:17 And sometimes we have difficulty, for example, in, for example, holding a selection process, I need a not the people here in our municipality also need to understand what a notice is, what this notice is requesting, and so, the vast majority of our population here, has a high school education, so sometimes this bureaucratization ends up complicating, so that we have, ends up contributing to our having these difficulties.
Mas, em específico, o Criança Feliz, ele não é um estágio curricular. Então, é uma coisa assim que, infelizmente para eles, eles até ficam meio desestimulados quando eles vêm para cá, porque eles não podem contar como horas, né? Então, mas aí, para estimulá-los, a gente, a contratação é de 30 horas, né? Eles trabalham 30 horas. Quando eles têm que fazer as 10 horas curricular, a gente insere eles no município mesmo. Então, aí a gente acaba, a gente tem estagiário lá do Criança Feliz, que está estagiando dentro do CRAS	14:6 But specifically, Criança Feliz, it is not a curricular internship. So, it's something that, unfortunately for the they even get a little discouraged when they come here, because they can't count it as hours, right? So, to encourage them, we, the hiring is for 30 hours, right? They work 30 hours. When they have to do the 10 curricu hours, we put them in the municipality itself. So, we end up, we have interns from Criança Feliz, who are interni at the CRAS
acho que acaba sendo um diferencial esse acúmulo de conhecimento que eles já têm enquanto estagiários. É claro que a carga horária também é uma dificuldade nossa, porque o estagiário trabalha 30 horas semanais, então o tempo já fica mais escasso. Mas esse agregado de conhecimento que ele tem acaba facilitando um pouco o trabalho deles com as famílias	15:7 I think this accumulation of knowledge they already have as interns ends up being a differential. Of course the workload is also a difficulty for us because the intern works 30 hours a week, so the time is already scarce. But this added knowledge they have ends up making their work with families a little easier.
ela não leva brinquedo não, aí eu faço em casa; ele brinca muito com chaves, coisas que faz barulho; ele tem uma bola de plástico que ela sai rolando e vai correndo atrás dela	5:48 she doesn't bring toys, then I do it at home; he plays a lot with keys, things that make noise; he has a plas ball that rolls and he runs after it

Workforce Expectations	English Translation
Eu acho que agora estão pensando nessa possibilidade do visitador, ele mesmo registrar a visita dele, sobre a supervisão do gestor e até do órgão gestor, ele ter acesso livre para ele fazer até mesmo uns relatórios, colocar observação, para às vezes pode mudar de visitador um ou outro que vim, estar lá, informatizado. Eu acho que isso seria essencial também. Era uma coisa que tem que se pensar. Agora, do mais, um recurso que com certeza se viesse mais, seria muito bem utilizado.	14:15 I think they are now considering this possibility for the visitor to register their visit themselves, under the supervision of the manager and even the managing agency, to have free access to even make some reports, add observations, so that sometimes a change of visitors, one or another that comes, can be there, computerized. I think this would be essential too. It's something that needs to be considered. Now, besides that, a resource that if it came in more would certainly be very well utilized.
O vínculo deles está contratado. Por período que estamos, da pandemia, não pode fazer seletiva. Foi de pessoas que mais têm pedagogia. A gente sugeriu mais que já têm um conhecimento para lidar com crianças. E já teve, dentro desse período, quatro profissionais que desistiram. Porque é um desafio para quem está aqui assumindo e um desafio para quem vem enfrentar. Num preparo psicológico, que eles não tinham de chegar em uma casa, está faltando alimento, ou não tem uma fralda, a criança não tinha uma fralda, ou estava toda assada, ou não tinha um colchão. Então, não ficava. Quatro pessoas já desistiram.	13:67 Their bond is contracted. During the pandemic period, no selection can be made. It was from people who have more pedagogy. We suggested more that already have knowledge to deal with children. And there have been, within this period, four professionals who gave up. Because it's a challenge for those who are here taking over and a challenge for those who come to face it. In a psychological preparation, which they didn't have to arrive at a house, lacking food, or not having a diaper, the child didn't have a diaper, or was all rashy, or didn't have a mattress. So, they didn't stay. Four people have already given up.
eu acho que tem um desafio, e pode estar correndo risco. Eu, no caso, foi até bem difícil passar pra mim, não sei se há problema falar disso aqui, só que eu fui fazer uma visita uma vez e um cara tentou abusar de mim, ele me agarrou, fiquei super abalada, consegui me defender ali na hora, e eu acho que um desafio, eu acho que o meu medo é esse, entendeu?	19:7 I think there's a challenge, and there may be a risk. In my case, it was even quite difficult for me to pass through, I don't know if there's a problem talking about it here, but once I went on a visit and a guy tried to abuse me, he grabbed me, I was very shaken, I managed to defend myself right there, and I think a challenge, I think my fear is this, you know?
E toda sexta-feira a gente tem tipo uma mini-formação. Por que mini-formação? Ali nós vamos fazer saber o que está acontecendo, qual é a dúvida, qual é a incerteza, qual é a insegurança . O visitador fala, ó, eu estou me emocionando naquela família, eu chego lá e me dá vontade de chorar quando eu chego em casa porque a situação é assim, aquilo, outro. Então a gente tem esse cuidado para treinar.	19:10 And every Friday we have like a mini-training. Why mini-training? There we will find out what's happening, what the doubts are, what the uncertainties are, what the insecurities are. The visitor says, oh, I'm getting emotional with that family, I get there and feel like crying when I get home because the situation is like that, another. So we take care to train them.
Nós temos regiões que distam 35 quilômetros, por exemplo, e que vai ter ônibus duas vezes no dia, no máximo. E que tem toda uma dificuldade de terreno. Então é o transporte, é o terreno, porque é um terreno que alaga, que atola, tem momentos em que você só pode andar pela praia, então você é dependente de maré.	7:9 We have regions that are 35 kilometers away, for example, and that will have a bus only twice a day, at most. And there's a whole difficulty with terrain. So it's transportation, it's the terrain, because it's terrain that floods, gets stuck, there are times when you can only walk along the beach, so you're dependent on the tide.
ele não foi pensado na característica do município, né, igual aquele assunto das 45 minutos da visita. O que esperava-se, né, acho que quando criou-se o programa, que uma casa fosse do lado da outra, que o visitador conseguisse atender, hoje cada visitador nosso atende 25 famílias, que é a meta deles, cinco famílias no dia seria. Esperava-se que essas famílias morassem todas pertas, imagino, né, para dar o tempo necessário para as visitas, mas não funciona desse jeito	8:1 It wasn't designed with the municipality's characteristics in mind, right, like that issue of the 45-minute visit. What was expected, I think when the program was created, was that one house would be next to the other, that the visitor could attend to, today each of our visitors attends to 25 families, which is their goal, five families a day would be. It was expected that these families all lived nearby, I imagine, to give the necessary time for the visits, but it doesn't work that way
a Água Branca, a gente conta com essa dificuldade do território, são, a maior parte da população é zona rural, né? Mas temos o transporte, temos o motorista, faz com o carro, aí a gente faz o cronograma, né? Para levar as visitadoras.	11:2 Água Branca, we face this difficulty with the territory, most of the population is rural, right? But we have transportation, we have the driver, he uses the car, then we make the schedule, right? To take the visitors.
não abrange a área rural, só a área urbana. Nós temos essa dificuldade com a... Nós, assim, que falo durante esses seis meses, porque eu que vejo a necessidade, que quando eu trabalhava, eu trabalhava na área da enfermagem com essa zona rural, que é o que mais acho que precisa, que é o quilombola, o assentamento, eu fazia um trabalho já com essa equipe do PSF que eu trabalhava. Então, quando eu cheguei aqui no social, já era dividido, só tinha a zona urbana, não tinha a zona rural. E eu vejo a necessidade... Fica sem um acompanhamento, mas também vejo um desafio quanto ao meio de transporte. Nós não temos. Nós temos um carro para atender o CRAS, o social, que é o agendamento, encaminhamento para o INSS, para as visitas, quando elas saem na zona rural, e também o conselho tutelar. Então, nós não temos recurso para fazer um trabalho para atingir a zona rural.	13:66 It doesn't cover the rural area, only the urban area. We have this difficulty with... We, like, I say during these six months, because I see the need, that when I worked, I worked in the nursing area with this rural area, which I think needs more, which is the quilombola, the settlement, I already did some work with this PSF team I worked with. So, when I got here in social work, it was already divided, it only had the urban area, there was no rural area. And I see the need... It goes without monitoring, but I also see a challenge regarding transportation. We don't have it. We have a car to serve the CRAS, the social work, which is scheduling, referral to the INSS, for visits, when they go out in the rural area, and also the guardianship council. So, we don't have the resources to do work to reach the rural area.
Nós temos extensão de 74 quilômetros de praia, nós não temos um núcleo, vamos assim dizer. Então, eu tenho balneários que são muito distantes, que a gente precisa de utilizar o nosso veículo. Dependemos de maré, porque se a maré está alta, a gente não consegue chegar nesse balneário, a gente precisa ir pelo continente, aí demora mais algumas horas para chegar lá, e são pessoas que ficam mais afastadas da gente também.	0.590277778 We have a coastline extension of 74 kilometers, we don't have a core, let's put it that way. So, I have resorts that are very distant, where we need to use our vehicle. We depend on the tide, because if the tide is high, we can't reach that resort, we need to go by land, then it takes a few more hours to get there, and these are people who are further away from us too.
então, assim, o nosso território rural, ele é muito extenso, então, assim, só de aldeias nós temos mais de 90 aldeias e mais seis assentamentos, então, assim, a gente tá fazendo atendimento de algumas famílias e algumas aldeias mais próximas, mas, assim, já tem famílias de outras aldeias que querem ser acompanhadas, mas a gente tem essa dificuldade, porque agora, nesse período que não tá chovendo, até que a gente consegue fazer algumas visitas, mas no período de chuvoso é bem complicado, em algumas regiões, para a gente chegar até lá, e aí, para a gente, a gente não tem recurso suficiente para alocar ou fazer a aquisição de uma caminhonete, a gente só aloca um carro pequeno e, devido à situação das estradas que a gente tem, aí é bem complicado para a gente atender,	14:21 So, like, our rural territory, it's very extensive, so, like, just from villages we have over 90 villages and another six settlements, so, like, we're attending to some families and some villages closer, but, like, there are already families from other villages that want to be followed, but we have this difficulty, because now, in this period when it's not raining, we can manage to make some visits, but in the rainy period it's really complicated, in some regions, for us to get there, and then, for us, we don't have enough resources to allocate or acquire a pickup truck, we only allocate a small car and, due to the condition of the roads we have, then it's really complicated for us to attend,
É um distrito que fica bem distante daqui, né, são cerca aí de, são quase 30 quilômetros de distância, numa estrada bem ruim, né. Então a gente colocou uma visitadora exclusiva para atender a demanda desse distrito, né	18:94 It's a district that is quite far from here, right, it's about, it's almost 30 kilometers away, on a very bad road, right. So we put an exclusive visitor to meet the demand of this district, right.
O nosso município aqui tem uma extensão territorial um pouco extensa, e as famílias moram em comunidades muito longe, né? De difícil acesso. Então, essa é a nossa maior dificuldade que a gente tem, é o acesso às famílias, né? Seja por questão de distância, seja por questão de comunicação. Muitas famílias a gente não consegue localizar na casa. A gente não consegue fazer o atendimento..	18:101 Our municipality here has a slightly extensive territorial extension, and families live in very distant communities, right? With difficult access. So, that's our biggest difficulty, is access to families, right? Whether it's because of distance or communication issues. Many families we can't locate at home. We can't provide service.
Só que eu acabo que eu faço duas coisas ao mesmo tempo, né, eu tenho metade do meu tempo dedicado ao programa e metade dedicado à gestão do sistema único, né, que é da assistência social. Então, acaba que, às vezes, essa parte da gestão necessita de mais uma atenção e eu não consigo me dedicar tanto ao programa.	18:96 But then I end up doing two things at the same time, right, I have half of my time dedicated to the program and half dedicated to managing the single system, right, which is social assistance. So, sometimes this management part needs more attention and I can't dedicate myself so much to the program.
