# Supplementary material for: Implementation barriers of Brazil’s national home visitation program for early childhood development: A qualitative evaluation
Source: PLOS Glob Public Health. 2026 Apr 10;6(4):e0005203. doi: 10.1371/journal.pgph.0005203 (PMC13068277; doi:10.1371/journal.pgph.0005203)
Supplement: S4 File — (DOCX) [file pgph.0005203.s004.docx]

Supplement 4. Corresponding Home Visiting Workforce Needs Assessment (HVWNA) and Consolidated Framework for Implementation Research (CFIR) domains and definition

| **HVWNA construct (definition)** | **Related CFIR construct (definition)** |
| --- | --- |
| **Workforce Expectations** – Reflects how clearly the program specifies expected HV tasks, how competencies/standards inform training and professional development, and how recruitment/hiring processes identify qualified candidates. | **Inner Setting** – The organization’s internal context, including culture, climate, resources, and readiness for change. |
| **Curricula, Materials, and Resources** – Reflects the accessibility and quality of curricula, materials, and resources provided to HVs and families. | **Innovation Characteristics** – Features of the intervention itself (e.g., evidence strength, complexity, cost) that influence implementation. |
| **Training, Supervision, and Career Development** – Reflects the accessibility/relevance of training and supervision for HVs and supervisors, plus career advancement opportunities. | **Process of Implementation** – The activities that drive implementation—planning, engaging stakeholders, executing, and reflecting/evaluating. |
| **Workforce Conditions** – Reflects compensation, workforce recognition mechanisms, workload, and organizational culture shaping day-to-day delivery. | **Inner Setting** – The organization’s internal context, including culture, climate, resources, and readiness for change. |
| **Program Design** – Reflects key design aspects including target population, intensity of services, and content. | **Innovation Characteristics** – Features of the intervention itself (e.g., evidence strength, complexity, cost) that influence implementation. |
| **Enabling Environment** – Reflects the overall operating context, including division of responsibilities across government levels, buy-in, available funding, and leadership capacity. | **Outer Setting** – External context and pressures—such as policies, funding, and stakeholder needs—shaping implementation. |
| **Quality Monitoring and Assurance** – Reflects how comprehensive monitoring/QA systems are upheld, implementation capacity, and how monitoring is used to sustain the program. | **Process of Implementation** – The activities that drive implementation—planning, engaging stakeholders, executing, and reflecting/evaluating. |
