# Supplementary material for: Implementation barriers of Brazil’s national home visitation program for early childhood development: A qualitative evaluation
Source: PLOS Glob Public Health. 2026 Apr 10;6(4):e0005203. doi: 10.1371/journal.pgph.0005203 (PMC13068277; doi:10.1371/journal.pgph.0005203)
Supplement: S5 File — (DOCX) [file pgph.0005203.s005.docx]

Supplement 5. Integrated HVWNA–CFIR crosswalk linking each barrier theme to HVWNA dimension and CFIR domain

| **Barrier theme (summary statement)** | **HVWNA dimension** | **CFIR domain** |
| --- | --- | --- |
| Lack of a structured curriculum and clear guidance on age-appropriate home-visit activities | Curricula, Materials, and Resources | Innovation Characteristics |
| Difficulty adapting home-visit content to diverse local contexts and cultural realities | Curricula, Materials, and Resources | Innovation Characteristics |
| Insufficient guidance on adapting visits/content for children with disabilities | Curricula, Materials, and Resources | Innovation Characteristics |
| Home visitors and/or families lack household materials/toys needed to implement planned activities | Curricula, Materials, and Resources | Innovation Characteristics |
| Families desire longer/more frequent visits, but competing responsibilities and logistics limit feasible intensity | Program Design | Innovation Characteristics |
| Hiring/contracting constraints (including restricted modalities) contribute to underqualified staffing and high turnover | Workforce Expectations | Inner Setting |
| PCF implementation is not mandatory; adherence depends on local political will | Enabling Environment | Outer Setting |
| Delays in registering/enrolling newborns delay access to PCF services during a critical period | Enabling Environment | Outer Setting |
| Many families lack adequate materials/toys to practice activities between visits | Curricula, Materials, and Resources | Innovation Characteristics |
| Long distances between households hinder delivering visits with required frequency and duration | Workforce Conditions | Inner Setting |
| Transportation for home visits is not consistently provided or available | Workforce Conditions | Inner Setting |
| Funding does not account for territorial access (distance/difficulty), limiting service to remote/traditional communities | Enabling Environment | Outer Setting |
| Working with families in extreme vulnerability creates emotional burden for home visitors with limited supports | Workforce Conditions | Inner Setting |
| High turnover disrupts onboarding and makes it difficult for new staff to receive effective training | Workforce Conditions | Inner Setting |
| Supervision is inconsistently structured and supervisors have competing duties, limiting coaching and oversight | Training, Supervision, and Career Development | Inner Setting |
| Home visitors have difficulty delivering effective visits for children with disabilities and in traditional communities (skills/contextual competence gaps) | Training, Supervision, and Career Development | Inner Setting |
| High cultural/linguistic diversity requires substantial adaptation of protocols and materials | Curricula, Materials, and Resources | Innovation Characteristics |
| Family-selection processes incentivize prioritizing households closer to the municipal center, excluding remote families | Program Design | Innovation Characteristics |
| Cascade training leaves some supervisors underprepared (limited pedagogic background), weakening downstream training quality | Training, Supervision, and Career Development | Inner Setting |
| Training content insufficiently covers child development, pedagogy, and program structure; implementers request expert-led modules | Training, Supervision, and Career Development | Inner Setting |
| Training agenda and visit guide provide inadequate guidance for delivering visits for children with disabilities | Training, Supervision, and Career Development | Inner Setting |
| Monitoring systems remain partly paper-based, increasing burden and reducing usability | Quality Monitoring and Assurance | Process |
| Home visitors struggle to understand and administer the child-development monitoring instrument | Quality Monitoring and Assurance | Process |
| Implementers receive limited/no training on monitoring instruments (including the electronic system); monitoring module needed | Quality Monitoring and Assurance | Process |
